# An ACAT inhibitor suppresses SARS-CoV-2 replication and boosts antiviral T cell activity

**DOI:** 10.1371/journal.ppat.1011323

**Published:** 2023-05-03

**Authors:** Peter A. C. Wing, Nathalie M. Schmidt, Rory Peters, Maximilian Erdmann, Rachel Brown, Hao Wang, Leo Swadling, COVIDsortium Investigators, Joseph Newman, Nazia Thakur, Kaho Shionoya, Sophie B. Morgan, Timothy SC Hinks, Koichi Watashi, Dalan Bailey, Scott B. Hansen, Andrew D. Davidson, Mala K. Maini, Jane A. McKeating

**Affiliations:** 1 Chinese Academy of Medical Sciences Oxford Institute, University of Oxford, Oxford, United Kingdom; 2 Nuffield Department of Medicine, University of Oxford, Oxford, United Kingdom; 3 Division of Infection and Immunity and Institute of Immunity and Transplantation, UCL, London, United Kingdom; 4 School of Cellular and Molecular Medicine, University of Bristol, Bristol, United Kingdom; 5 UCL Queen Square Institute of Neurology, London, United Kingdom; 6 Departments of Molecular Medicine and Neuroscience, The Scripps Research Institute, San Diego, California, United States of America; 7 Skaggs Graduate School of Chemical and Biological Sciences, The Scripps Research Institute, San Diego, California, United States of America; 8 The Pirbright Institute, Woking, United Kingdom; 9 Department of Virology II, National Institute of Infectious Diseases, Tokyo, Japan; 10 Department of Applied Biological Science, Tokyo University of Science, Noda, Japan; 11 Research Centre for Drug and Vaccine Development, National Institute of Infectious Diseases, Tokyo, Japan; 12 Respiratory Medicine Unit and National Institute for Health Research Oxford Biomedical Research Centre, Nuffield Department of Medicine, Experimental Medicine, University of Oxford, Oxford, United Kingdom; Ruhr-Universitat Bochum, GERMANY

## Abstract

The severity of disease following infection with SARS-CoV-2 is determined by viral replication kinetics and host immunity, with early T cell responses and/or suppression of viraemia driving a favourable outcome. Recent studies uncovered a role for cholesterol metabolism in the SARS-CoV-2 life cycle and in T cell function. Here we show that blockade of the enzyme Acyl-CoA:cholesterol acyltransferase (ACAT) with Avasimibe inhibits SARS-CoV-2 pseudoparticle infection and disrupts the association of ACE2 and GM1 lipid rafts on the cell membrane, perturbing viral attachment. Imaging SARS-CoV-2 RNAs at the single cell level using a viral replicon model identifies the capacity of Avasimibe to limit the establishment of replication complexes required for RNA replication. Genetic studies to transiently silence or overexpress ACAT isoforms confirmed a role for ACAT in SARS-CoV-2 infection. Furthermore, Avasimibe boosts the expansion of functional SARS-CoV-2-specific T cells from the blood of patients sampled during the acute phase of infection. Thus, re-purposing of ACAT inhibitors provides a compelling therapeutic strategy for the treatment of COVID-19 to achieve both antiviral and immunomodulatory effects.

**Trial registration**: NCT04318314.

## Author summary

Cholesterol metabolism is a key element in both viral replication and immune cell signalling. We show that Avasimibe, an inhibitor of the cholesterol esterification enzyme Acyl-CoA:cholesterol acyltransferase (ACAT), potently inhibits SARS-CoV-2 entry and viral RNA replication. Importantly, Avasimibe boosts the expansion of functional SARS-CoV-2-specific T cells isolated from the blood of patients sampled during the acute phase of infection. Our findings show that Avasimibe has combined antiviral and T cell boosting properties in acute SARS-CoV-2 infection. This study reinforces an emerging picture of the importance of cholesterol metabolism in viral replication and T cell immunity, providing new insights on intrinsic anti-viral pathways that may be applicable to other viral pathogens.

## Introduction

SARS-CoV-2 is a global health issue associated with over 600 million infections and 6 million deaths by October 2022 [1]. Preventive vaccines have reduced morbidity and mortality [2,3] and several direct-acting antiviral drugs are now licensed for the treatment of SARS-CoV-2 infection such as Remdesivir and Ritonavir-boosted Nirmatrelvir [4]. However, additional therapeutic strategies for unvaccinated subjects or those with breakthrough infections are still needed. Several studies report a role for SARS-CoV-2-specific T cells in the early control of viraemia associated with mild, asymptomatic or even abortive infection [5–9]. In contrast, features of T cell dysfunction and exhaustion including a loss of effector function and expression of exhaustion markers such as programmed death-1 (PD-1) have been reported in SARS-CoV-2 infection, particularly in patients with severe disease [10–12], suggesting that approaches to restore T cell functionality may be beneficial. To the best of our knowledge there are currently no agents that show both direct antiviral and immune boosting activity against SARS-CoV-2 infection.

Metabolic syndrome and hyperlipidaemia have been associated with a poorer outcome of SARS-CoV-2 infection and cholesterol-lowering HMG-CoA-reductase inhibitors (statins) may improve COVID-19 survival, highlighting the potential of targeting cholesterol metabolism as a treatment strategy [13–15]. Cholesterol is a key component of cellular membrane lipids regulating curvature, fluidity and the formation of microdomains or lipid rafts in the plasma membrane that are sites of receptor signalling [16]. In immune cells, cholesterol availability, uptake and utilization are linked to immune function and shape antiviral responses [15,17,18].

Cholesterol homeostasis is integral to replication of a wide range of viruses, impacting multiple steps in the viral life cycle including entry, replication, assembly and egress [19]. Recent studies have identified a role for cholesterol in SARS-CoV-2 particle infectivity, syncytia formation and genome replication [20–22]. The cell membrane is a dynamic and structured environment, heavily influenced by the relative cholesterol content which comprises cholesterol and sphingolipid rich microdomains or lipid rafts. Beyond key roles in T cell signalling, lipid rafts regulate coronavirus entry, mediating attachment of infectious bronchitis virus and porcine delta coronavirus [23,24]. Importantly, association of angiotensin converting enzyme 2 (ACE2) with monosialotetrahexosylganglioside1 (GM1) in lipid rafts is essential for SARS-CoV-1 infection [25]. ACE2 can traffic between GM1 and phosphatidylinositol 4,5-bisphosphate (PIP_2_) clusters, which are commonly associated with disordered regions of the cellular membrane, in a cholesterol-dependent manner [26]. Anaesthetics and hydroxychloroquine have been shown to perturb ACE2 localization with GM1 and PIP_2_ clusters, inhibiting SARS-CoV-2 entry [27]. In addition, the SARS-CoV-2 spike protein can bind cholesterol in high density lipoprotein (HDL) particles, that mediate virus entry through scavenger receptor B type 1 mediated uptake of HDL within the context of ACE2 co-expression [28].

In addition to viral entry, recent genetic screens have implicated cholesterol homeostasis as an essential process in coronavirus RNA replication [29–34]. Like most positive stranded RNA viruses, SARS-CoV-2 establishes replication organelles within double-membrane vesicles (DMVs), which provide a platform for viral replication complexes and protection from innate immune surveillance [35]. DMVs are enriched in cholesterol-rich lipid rafts and semi-synthetic cholesterol derivatives such as Oxy210 attenuate DMV formation reducing formation of viral replication complexes [36]. Moreover, the cholesterol transport and biosynthesis inhibitor U18666A blocks feline coronavirus replication by dysregulating the cholesterol transporter Niemann-Pick type C1 receptor which is implicated in the life cycle of several viruses [37–40].

Acyl-CoA:cholesterol acyltransferase (ACAT, also known as sterol O-acyltransferase, SOAT) esterifies free cholesterol; previous work demonstrated that pharmacological inhibition of ACAT reduced hepatitis B and C virus replication [41,42], whilst enhancing antiviral and anti-tumour T cell responses [42,43]. We previously reported that ACAT inhibition induced metabolic reprogramming to boost the exhausted T cell response that is characteristic of chronic hepatitis B and hepatocellular carcinoma [42]. We found that cholesterol-rich microdomains required for T cell synapse formation and antigen recognition were reduced in exhausted T cells expressing high levels of PD-1 (PD-1^hi^) and ACAT inhibition restored these properties, suggesting it may provide beneficial effects on the activated PD-1^hi^ antiviral T cells in acute SARS-CoV-2 infection. Thus, we hypothesized that modulation of cholesterol metabolism by ACAT inhibitors such as Avasimibe (AVS) would inhibit SARS-CoV-2 replication and boost virus-specific T cells to control infection.

## Results

### Avasimibe inhibits SARS-CoV-2 pseudoparticle entry

**Fig 1:**
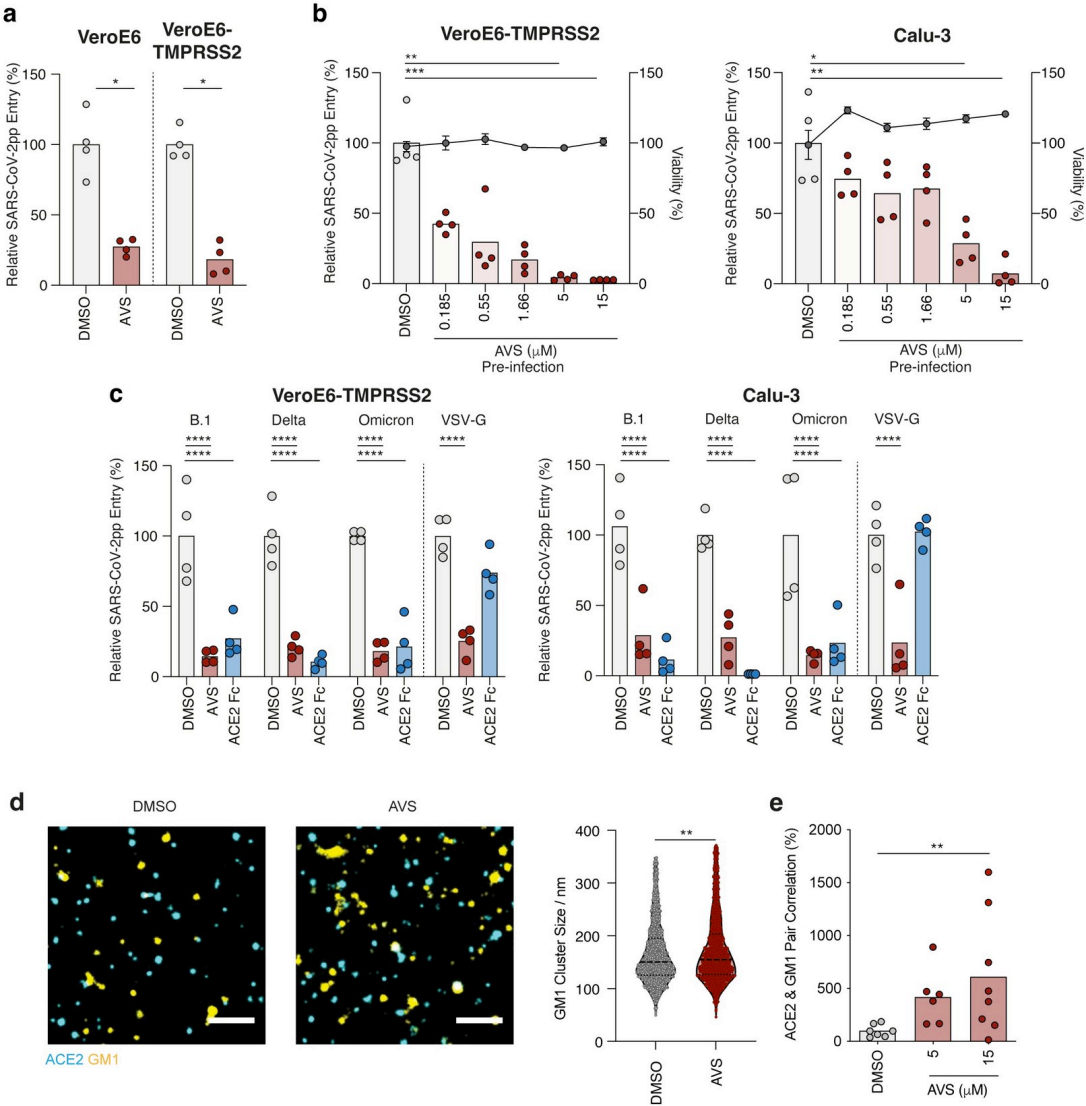
Avasimibe inhibits SARS-CoV-2 pseudoparticle entry. (a) VeroE6 and VeroE6-TMPRSS2 cells were treated with 10μM of Avasimibe (AVS) for 24h prior to infection with lentiviral pseudoparticles (pp) bearing the SARS-CoV-2 spike protein (VIC 01/20) and luciferase activity measured 48h post-infection. (b) Vero-TMPRSS2 (left) and Calu-3 (right) cells were pre-treated for 24h with AVS (red) or DMSO (light grey) and infected with SARS-CoV-2pp (VIC 01/20). Luciferase activity and cell viability (dark grey) were measured 48h post-infection and data are representative of n = 4 biological replicates. (c) Viral pp were generated bearing spike proteins from B.1, Delta, and Omicron variants of concern or VSV-G and used to infect VeroE6-TMPRSS2 (left) or Calu-3 (right) cells pre-treated with 10μM of AVS. As a control to evaluate ACE2-dependency of infection all pp were incubated with 1μg/mL of ACE2-Fc prior to infecting target cells. All data are normalized to mean of DMSO, and p values determined by ANOVA (Kruskal Wallis). (d) Representative images from dSTORM super resolution imaging of cholesterol dependent GM1 lipid cluster in plasma membrane. GM-1 cluster size (nm) determined by CTB staining assessed via dSTORM super resolution imaging (n = 1765–1966 clusters from n = 6 cells). (e) Quantification of GM-1 clusters and ACE2 by dSTORM imaging of VeroE6 cells treated with 5 or 15μM of AVS for 1h. Scale bar, 1μm. Significance was assessed by ANOVA or Mann Whitney T-test (d).

SARS-CoV-2 infection is initiated by the viral Spike protein binding to ACE2 at the cell surface. Subsequent cleavage by the transmembrane protease serine 2 (TMPRSS2) triggers fusion of viral and host membranes [44,45]. In addition, SARS-CoV-2 can infect cells lacking TMPRSS2, where particles are internalised by ACE2-dependent endocytosis with fusion occuring within endosomal vesicles [46]. To assess whether ACAT inhibition with AVS can regulate plasma membrane or endosomal viral fusion we used SARS-CoV-2 Spike protein (Victoria 01/20 strain) pseudotyped lentiviral particles (pp) expressing a luciferase reporter to infect VeroE6 cells that lack TMPRSS2, or cells engineered to over-express the protease (**S1A Fig**). Pre-treating cells with AVS reduced SARS-CoV-2pp infection of both VeroE6 and VeroE6-TMPRSS2 cells (**Fig 1A**). To evaluate whether AVS influenced the lentiviral promoter we transfected lentiviral DNA into VeroE6-TMPRSS2 cells and observed comparable luciferase activity in AVS and DMSO treated cells (**S1B Fig**), demonstrating that AVS has a minimal effect on lentiviral genome expression.

We extended our observations to Calu-3 cells, a lung epithelial cell line that expresses both ACE2 and TMPRSS2 [47], and observed a dose-dependent inhibition of SARS-CoV-2pp infection with an IC_50_ of 1.77μM compared to 0.23μM for Vero-TMPRSS2 cells and no detectable effect on cell viability (**Fig 1B**). The emergence of SARS-CoV-2 variants of concern (VOC) with altered Spike proteins such as Omicron that can evade vaccine protection prompted us to evaluate their sensitivity to ACAT inhibition. AVS inhibited pseudoparticles expressing B.1 (D614G), Delta and Omicron (BA.1) Spike proteins infecting VeroE6-TMPRSS2 and Calu-3 cells (**Fig 1C**). All SARS-CoV-2pp were neutralized with a saturating dose of ACE2-Fc, demonstrating ACE2-dependent infection. To evaluate whether this antiviral activity was dependent on endocytic trafficking we infected cells with pseudoparticles bearing Vesicular Stomatitis Virus G glycoprotein (VSV-G) that are internalized via clathrin-dependent endocytosis and fuse with endosomal membranes [48]. AVS reduced VSV-Gpp infection of both cell lines (**Fig 1C**), suggesting a role for ACAT in regulating endocytic trafficking pathways.

AVS has been reported to alter plasma membrane cholesterol levels, showing a reduction in hepatoma cells [49] and an increase in T cells [42,43], suggesting cell-type differences. Cholesterol levels in the AVS treated VeroE6 cells showed a modest but significant increase in free cholesterol with no change in total levels, consistent with a redistribution from cholesteryl esters stored in lipid droplets to unesterified membrane cholesterol (**S1C Fig**). Membrane cholesterol can cluster in lipid rafts, cholesterol- and glycosphingolipid-rich microdomains that can be identified by fluorescent-labelled cholera toxin B (CTB) subunit binding to GM1 and visualized by direct stochastical optical reconstruction microscopy (dSTORM). AVS increased the diameter of GM1-enriched domains in VeroE6 cells (**Fig 1D**), consistent with the increase in plasma membrane cholesterol. Next, we assessed whether the increase in GM1-enriched domains altered ACE2 localisation. Quantification of ACE2 and GM1 co-localisation by dSTORM imaging of VeroE6 cells revealed a significant increase with AVS treatment (**Fig 1E**). Collectively, these data highlight a role for AVS to inhibit SARS-CoV-2 uptake by regulating the association of ACE2 with GM1 lipid rafts.

### Avasimibe reduces SARS-CoV-2 attachment

**Fig 2:**
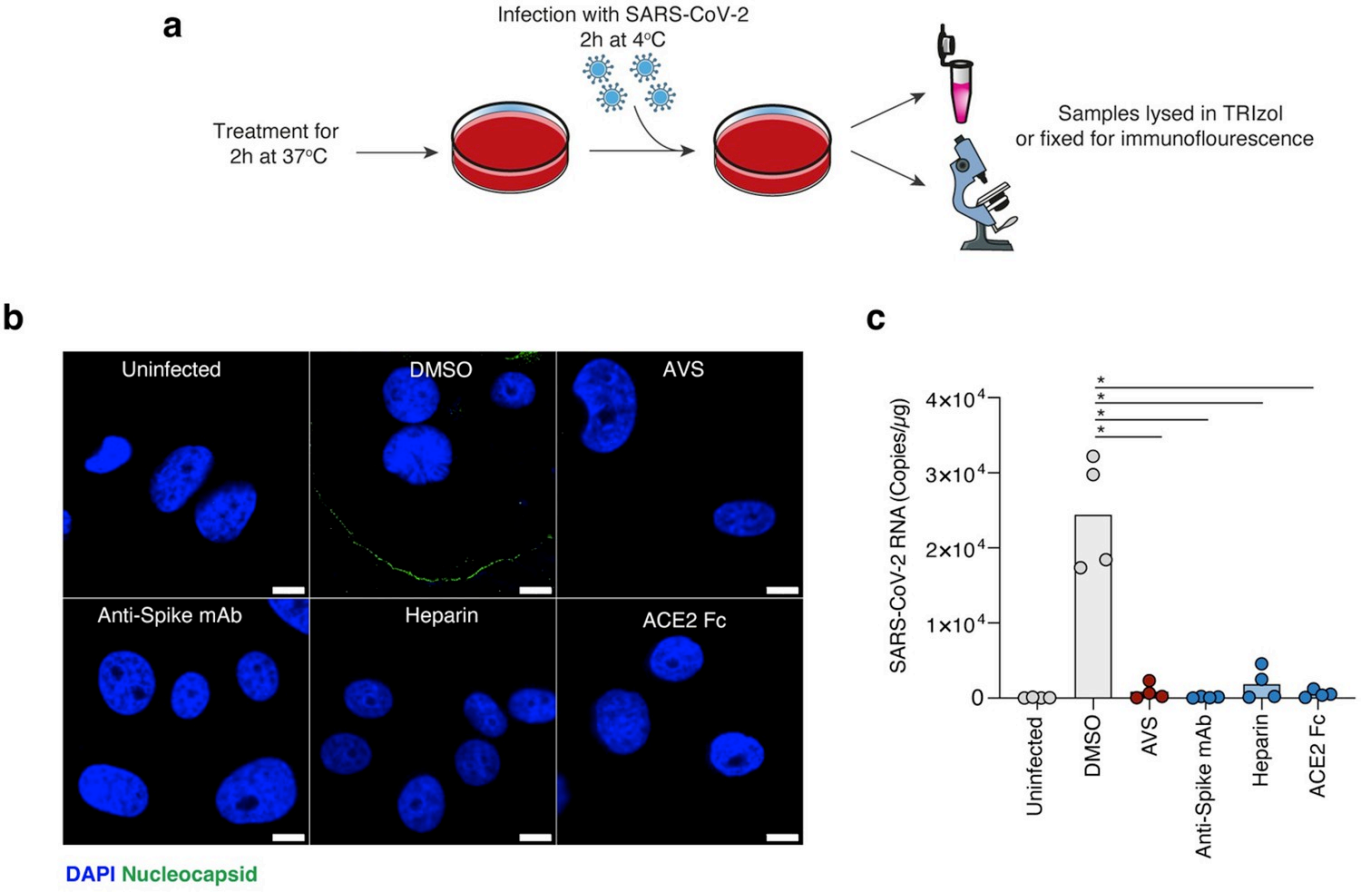
Avasimibe reduces SARS-CoV-2 attachment. a) Schematic of the binding assay. Chilled Vero-TMPRSS2 cells were pre-incubated with 10μM AVS, 1μg/mL Anti-spike mAb FI-3A, 100 U/mL Heparin or 1μg/mL ACE2-Fc for 2h at 37°C. Cells were infected with SARS-CoV-2 (MOI = 10) at 4°C for 2h, unbound virus was removed and samples either fixed for immunofluorescent staining of the viral nucleocapsid or lysed in TRIzol for qPCR quantification of viral RNA. Icons in figure were adapted from Servier Medical Art (https://smart.servier.com). (b) Representative confocal microscopy images of viral nucleocapsid staining with the indicated treatments. Images are taken at x192 magnification and scale bars represent 10μm. (c) Quantification of SARS-CoV-2 RNA copies by qPCR from matched samples depicted in (b). Data are derived from n = 4 biological replicates and significance determined by Kruskal-Wallis.

To further explore the mechanism underlying AVS inhibition of SARS-CoV-2pp infection we quantified viral binding to VeroE6-TMPRSS2 cells using a previously reported protocol [50]. AVS treated cells were inoculated with SARS-CoV-2 at an MOI of 10 whilst chilled on ice to allow virus binding but not uptake. Unbound virus was removed by multiple washes with cold PBS and cell bound virus measured by immunofluorescent staining of the SARS-CoV-2 nucleocapsid or qPCR quantification of viral RNA genomes (**Fig 2A**). AVS treated cells showed a reduction in nucleocapsid staining (**Fig 2B)** and viral RNA (**Fig 2C**) compared to control DMSO treated cells. Treating the viral inocula with a neutralizing anti-Spike mAb or ACE2-Fc prevented viral attachment, demonstrating Spike-ACE2 dependent particle attachment (**Fig 2C**). Furthermore, pre-treating the virus with heparin reduced virus attachment by competing for cell surface heparan-sulphate-proteoglycans, previously described as factors involved in SARS-CoV-2 attachment [51]. Together these data show that AVS treatment reduces the initial attachment of SARS-CoV-2 to the cellular membrane.

### Avasimibe inhibits SARS-CoV-2 infection and RNA replication

**Fig 3:**
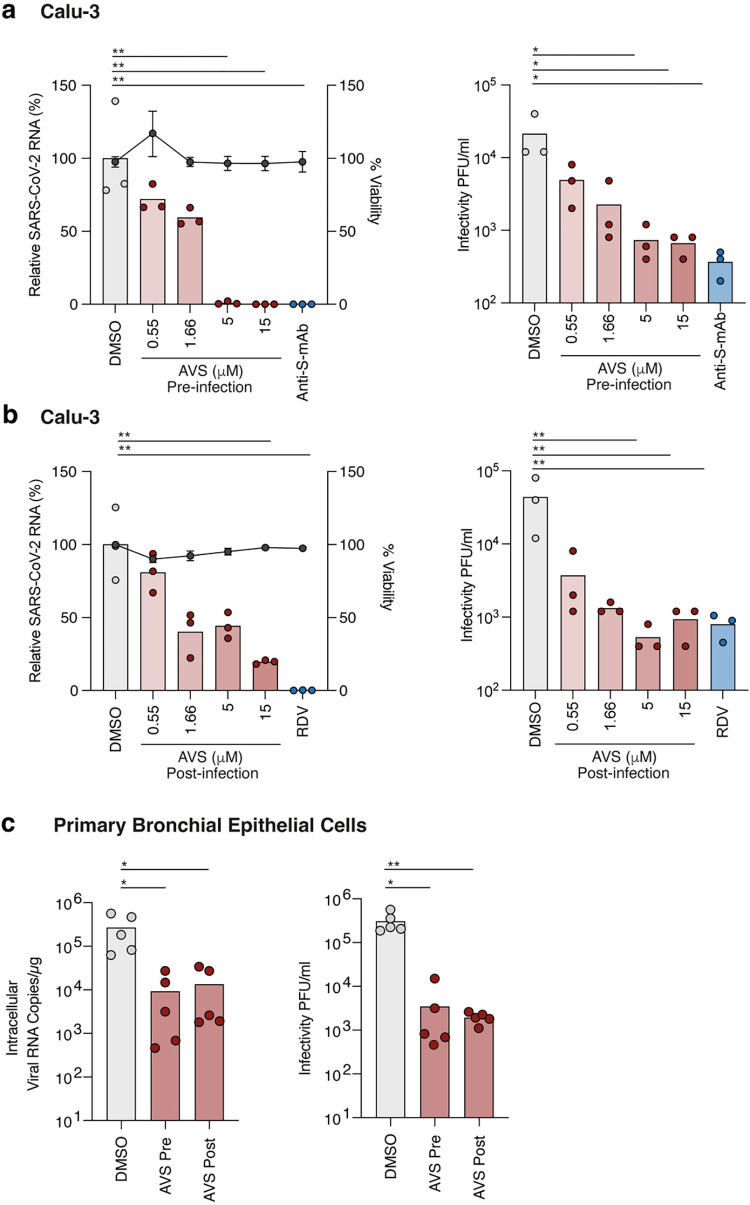
ACAT inhibition limits SARS-CoV-2 infection and replication. (a) Calu-3 cells were pre-treated with AVS for 24h prior to infection with SARS-CoV-2 (VIC 01/20) at an MOI of 0.01. Cells were harvested 24h post infection and intracellular viral RNA quantified by qPCR and infectious virus by plaque assay. As a control, cells were treated with 1μg/mL anti-Spike mAb FI-3A to neutralise infection. Data are representative of n = 3 biological replicates. (b) Calu-3 cells were infected with SARS-CoV-2 (MOI 0.01) for 2h, the inoculum removed, and cells treated with AVS. Cells were harvested 24h post infection and intracellular viral RNA quantified by qPCR and infectious virus by plaque assay. As an additional control, cells were treated with 1μM of RDV. Data are representative of n = 3 biological replicates. (c) Primary bronchial epithelial cells (PBEC) grown to air-liquid-interface were treated with 10μM of AVS either 24h pre- or 2h post infection of the apical surface with SARS-CoV-2 (MOI 0.1). Cultures were harvested 24h post infection and viral RNA quantified by qPCR and infectious virus shed from the apical surface by viral plaque assay. Data are representative of n = 5 donors and significance determined by ANOVA (Kruskal Wallis).

To determine whether our observations with lentiviral pp translate to authentic viral infection, we pre-treated Calu-3 cells with AVS prior to infection with SARS-CoV-2 (Victoria 01/20 strain) and observed a significant reduction in intracellular viral RNA and secreted infectious virus (**Fig 3A**). Notably, AVS showed comparable anti-viral activity to the neutralizing anti-Spike mAb FI-3A [52]. To examine whether ACAT regulates post-entry steps in the viral life cycle we treated Calu-3 cells with AVS following the establishment of infection and showed a similar reduction in viral RNA and infectious virus (**Fig 3B**). As a control for viral genome replication, cells were treated with the guanosine nucleoside analogue Remdesivir (RDV) (**Fig 3B**). We confirmed that treating infected VeroE6-TMPRSS2 with AVS showed a dose-dependent inhibition of intracellular viral RNA (**S2A Fig**). Finally, we assessed the effect of AVS on SARS-CoV-2 infection of human primary bronchial epithelial cells (PBEC) grown at air-liquid-interface to provide a more physiological model of infection. Treatment with AVS pre- or post-infection reduced viral RNA and infectious virus shed from the apical surface of the cultures (**Fig 3C**). Taken together, ACAT inhibition has an antiviral effect against SARS-CoV-2 infection and RNA replication.

**Fig 4:**
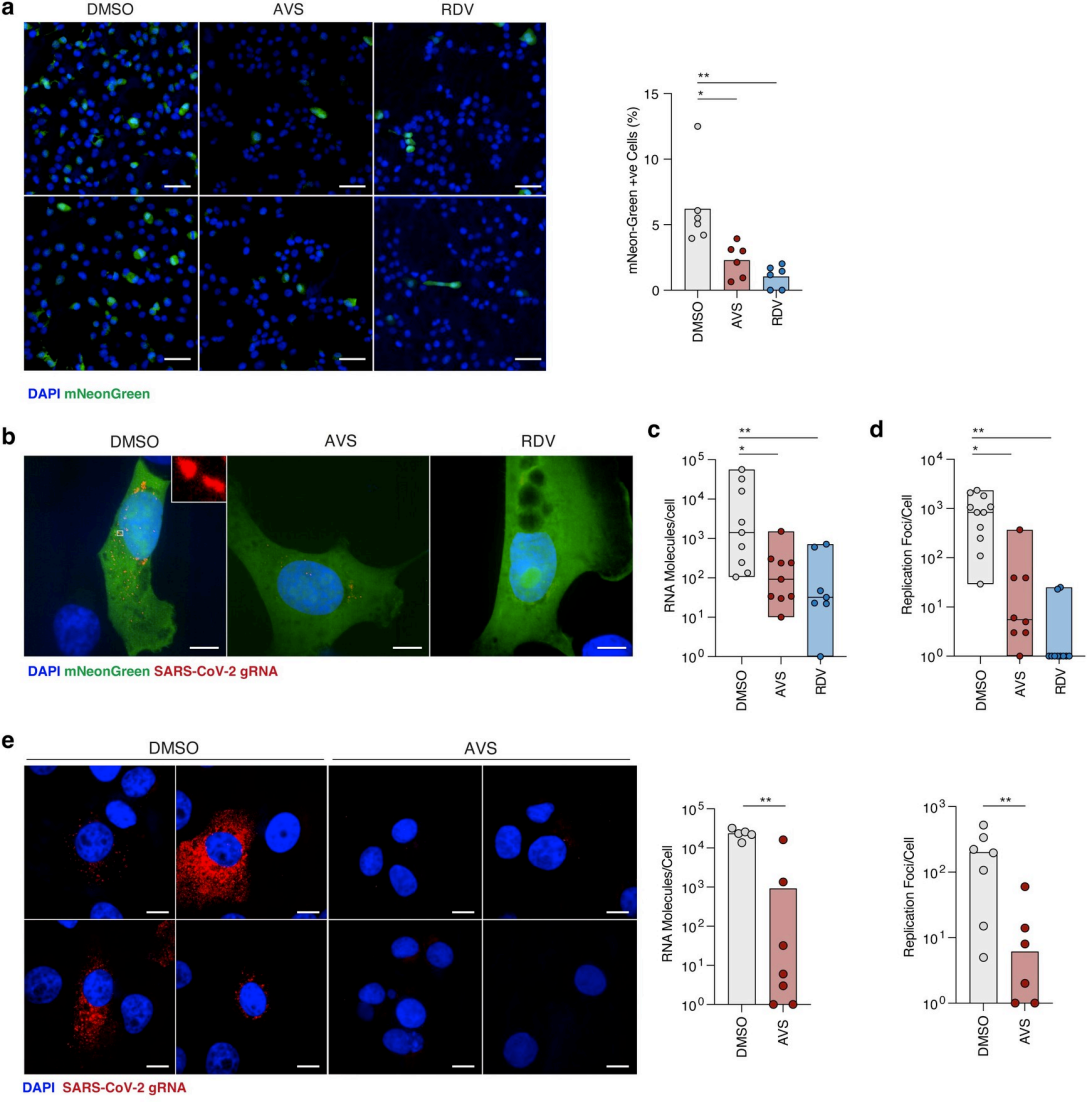
Avasimibe inhibits SARS-CoV-2 RNA replication. (a) Representative images of mNeonGreen positive cells from replicon RNA transfected cells harvested at 24h post-electroporation. Frequency of fluorescent cells was calculated by enumerating positive cells as a percentage of total cells in the field as determined by nuclei staining. Scale bar represents 100μm (b) Maximum z-projected confocal images of replicon expressing cells, treated with 10μM AVS or 1μM RDV and hybridised with fluorescent labelled probes to visualise replicon RNA. Scale bar represents 10μm. (c) BIGFISH quantification of gRNA counts in replicon transfected cells treated with AVS, RDV or control DMSO. Data represents the average RNA count per cell derived from at least 5–10 sample areas, where between 3–5 cells were quantified per area. (d) Quantification of replicon replication foci using smFISH cluster analysis. Data are average replication foci per cell derived from multiple sample areas as in (c). (e) Visualisation of SARS-CoV-2 RNA in Vero-TMPRSS2 cells infected with an MOI of 1 treated with or without AVS. Cells were infected for 1h, virus removed and treated with 10μM AVS for a further 5h before fixation for smFISH hybridisation. Images are maximum-z-projections from 4 representative sample areas. Viral gRNA counts and replication foci were quantified as in (c-d). Statistical significance was determined by Kruskal-Wallis. Scale bar represents 10μm.

To delineate the effect of AVS on SARS-CoV-2 entry and replication, we utilised a viral replicon system where the Spike and Membrane protein coding sequences are deleted to prevent the assembly of nascent viral particles. The SARS-CoV-2 replicon encodes both *Renilla* luciferase and mNeonGreen reporters in place of the membrane and ORF7a proteins respectively, which are expressed from sub genomic RNAs and can be used to monitor RNA replication [53]. Transient expression of the reporter proteins peaks at 24-36h post-transfection of RNA transcripts into permissive cells and is sensitive to RDV, providing a surrogate for SARS-CoV-2 replication [53]. Replicon RNA transcripts were electroporated into VeroE6-ACE2-TMPRSS2 cells and treated with AVS, RDV or control DMSO for 24h. RNA replication was initially assessed by quantifying mNeonGreen expressing cells and both treatments reduced the frequency of fluorescent cells (**Fig 4A**). We previously developed a single-molecule-fluorescent-*in-situ-*hybridisation (smFISH) technique to visualise SARS-CoV-2 RNAs within individual cells [54], allowing us to quantify total RNA and replication complexes within a cell. We applied this method to replicon transfected VeroE6-ACE2-TMPRSS2 cells using probes directed against the genomic RNA (gRNA) located in the ORF1A/B region. The probes detected foci of replicating RNA in mNeonGreen expressing cells, visible as distinct puncta distributed throughout the cytoplasm (**Fig 4B**). Quantifying replicon RNA molecules in AVS, RDV or DMSO treated cells showed a significant reduction with both drug treatments (**Fig 4C**). SARS-CoV-2 exploits host membranes to generate structured complexes where the viral genomes replicate during infection. Using the spatial resolution of smFISH, we quantified replication complexes, defined as spatially extended foci containing multiple gRNA molecules in the perinuclear region (**Fig 4B—**Inset), as previously reported [54]. We found a significant reduction in the number of replication foci with both AVS and RDV treatment compared to the DMSO control (**Fig 4D**).

To confirm our observations in the context of authentic viral infection, we infected VeroE6-TMPRSS2 cells with a high MOI of SARS-CoV-2 for 1h and, after extensive washing to remove the unbound viral inoculum, treated the cells with AVS. The infected cells were fixed and imaged at 6h post-infection, consistent with the establishment of viral replication complexes within the eclipse phase of the viral life cycle, allowing us to study primary RNA replication events [54]. During this short AVS treatment, we observed a significant reduction in both the number of viral RNA molecules and replication foci (**Fig 4E)**, collectively demonstrating a role for ACAT in regulating the establishment of SARS-CoV-2 replication complexes.

### A role for ACAT2 in SARS-CoV-2 infection

**Fig 5:**
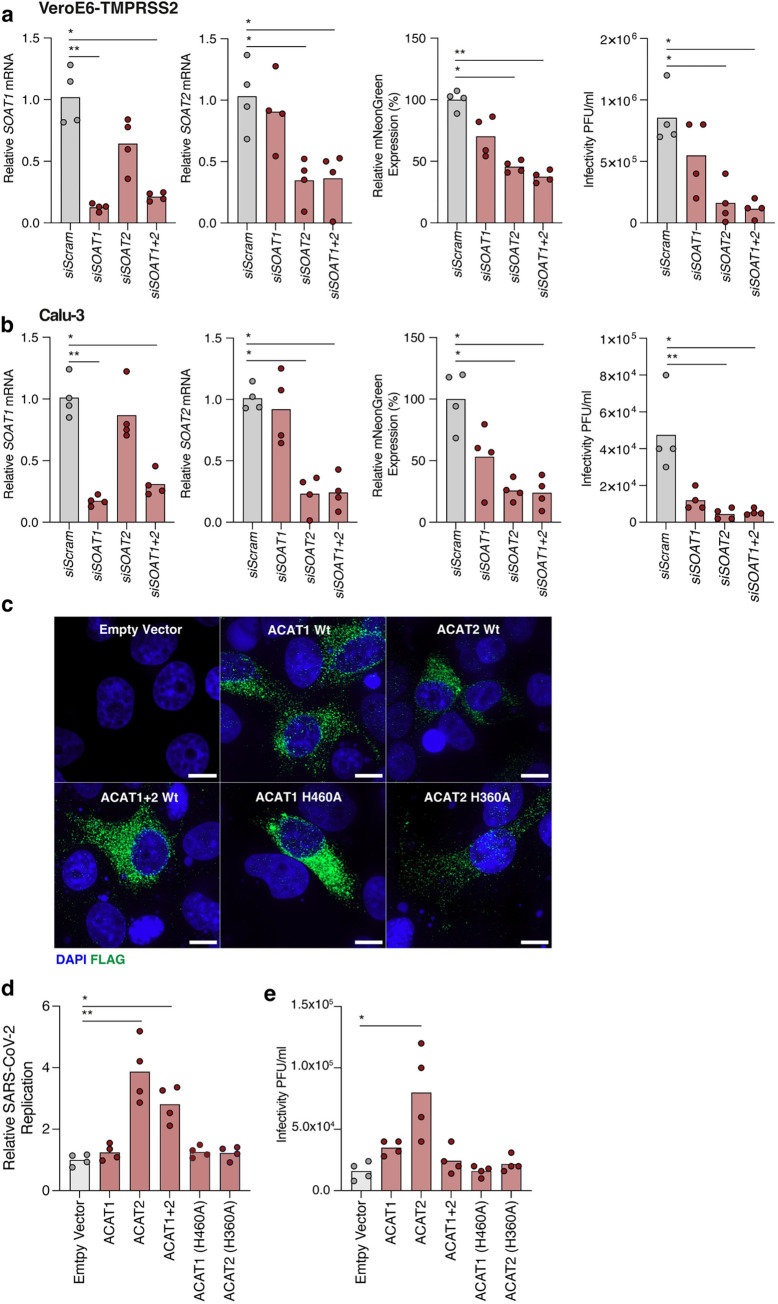
siRNA knockdown and overexpression of ACAT modulates SARS-CoV-2. (a) VeroE6-TMPRSS2 and Calu-3 (b) cells were transfected with siRNAs targeting *SOAT1* or *SOAT2*, respectively. Cells were infected with an infectious fluorescent SARS-CoV-2 reporter virus encoding mNeonGreen at an MOI of 0.01 and *SOAT1* and *SOAT2 m*RNA levels quantified by qPCR. mNeon-Green fluorescence was measured 24h post-infection and data plotted relative to the siRNA scramble control. Infectious virus from siRNA transfected cultures was assessed by plaque assay. Data is representative of n = 4 biological replicates. (c) Immunofluorescent imaging of VeroE6-TMPRSS2 cells transfected with FLAG-tagged ACAT1 and ACAT2 wild-type (Wt) or catalytically inactive mutants and stained with anti-FLAG. Images were taken at x192 magnification and scale bars represent 10μm. (d) VeroE6-TMPRSS2 cells, transfected with ACAT expression plasmids were infected with SARS-CoV-2_mNeonGreen at an MOI of 0.01 and fluorescence measured at 24h post infection. (e) Quantification of infectious virus secreted from cells in (d) by plaque assay. Data are representative of n = 4 biological replicates and statistical significance determined by Kruskal-Wallis.

ACAT has two isoforms, ACAT1 and ACAT2, encoded by *SOAT1* and *SOAT2* respectively. We confirmed gene expression of both isoforms in Vero-TMPRSS2, Calu-3 and PBEC cultures, finding higher levels of *SOAT1* expression in all three settings (**S2B Fig**). Analysis of previously published RNAseq data from the lung tissue of SARS-CoV-2 infected Golden Syrian Hamsters [55] showed a similar pattern of *Soat1* and *Soat2* expression (**S2C Fig**). To understand their respective roles in SARS-CoV-2 infection we transfected siRNAs either individually or in combination to knockdown (KD) *SOAT1* and *SOAT2* expression in VeroE6-TMPRSS2 or Calu-3 cells prior to infection. KD was confirmed by qPCR measurement of *SOAT1* and *SOAT2* mRNA respectively (**Fig 5A and 5B**). *SOAT1* KD had a limited effect on SARS-CoV-2 infection, whereas we observed a significant reduction in virus replication and infectious particles in *SOAT2* KD, either in isolation or in combination with *SOAT1* (**Fig 5A and 5B**). We confirmed this phenotype by overexpressing FLAG-tagged wild-type ACAT1 and ACAT2 along with catalytically inactive mutants in VeroE6-TMPRSS2 cells [56]. We confirmed endogenous expression of ACAT1 and ACAT2 protein expression in these cells (**S2D Fig**) and noted a comparable frequency of cells expressing exogenous wild-type or inactive ACAT1 (H460A) and ACAT2 (H360A) with similar patterns of cytoplasmic expression [56] (**Fig 5C**). Infection of cells overexpressing wild-type ACAT2, but not ACAT1, resulted in a significant increase in viral RNA and infectious particles (**Fig 5D and 5E**). Importantly this augmentation of viral replication was not observed in cells expressing the catalytically inactive ACAT2-H360A mutant, demonstrating that enzymic activity is necessary to promote viral replication. To consolidate these results we evaluated the antiviral activity of an ACAT1 inhibitor Nevanimibe [57,58], which showed a reduced antiviral activity (IC_50_ 27.4μM) compared to AVS (IC_50_ 4.60μM), consistent with a dominant role for ACAT2 in SARS-CoV-2 replication (**S2E Fig)**. In summary, these genetic data consolidate our earlier results with pharmacological inhibition of ACAT and collectively demonstrate a role for this acyltransferase in SARS-CoV-2 infection.

### Impact of Avasimibe on SARS-CoV-2-specific T cells

**Fig 6:**
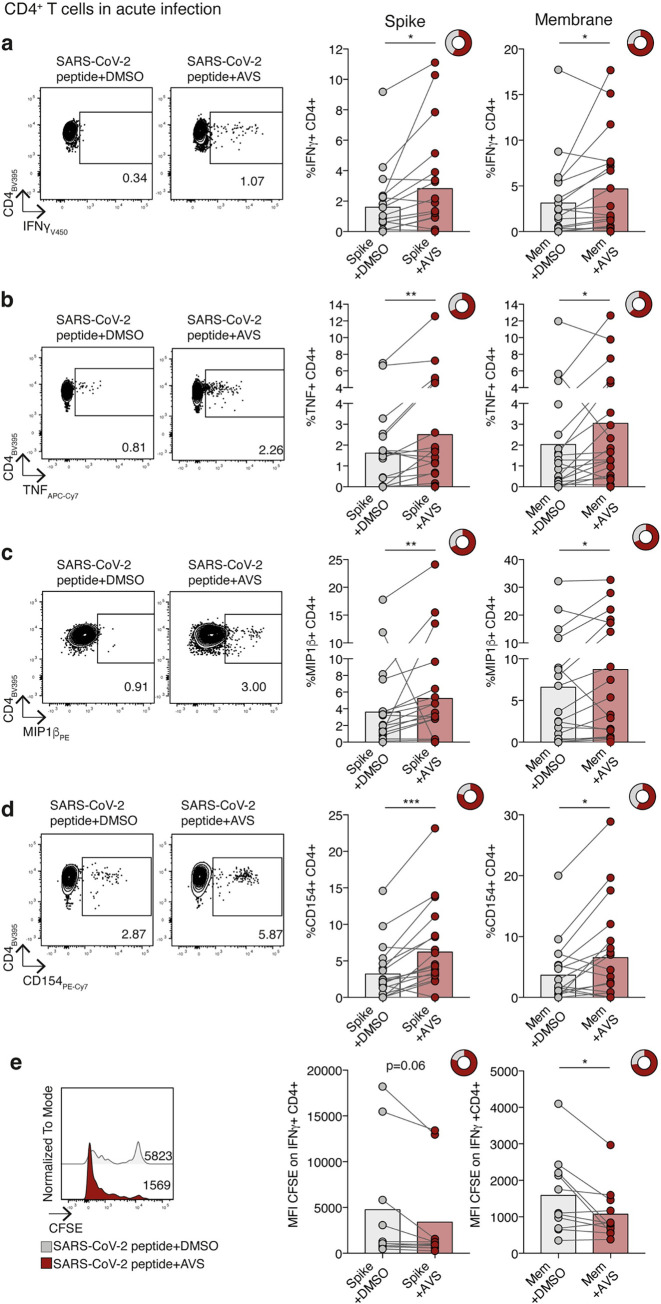
Effect of Avasimibe on SARS-CoV-2-specific CD4^+^ T cells in acute infection. (a-e) Human PBMC from donors with acute SARS-CoV-2 infection were stimulated with SARS-CoV-2 peptide pools (Spike and Membrane, Mem) and treated with Avasimibe (AVS) or DMSO for 8 days. SARS-CoV-2-specific cytokine production by CD4^+^ T cells was detected via flow cytometry. The cytokine production/CD154 expression in wells without peptide stimulation was subtracted to determine SARS-CoV-2-specific cytokine production/CD154 expression in summary data. Example plots and summary data for SARS-CoV-2 specific IFNγ (a), TNF (b), MIP1β (c) production and CD154 expression (d) by CD4^+^ T cells (n = 19). (e) Assessment of SARS-CoV-2-specific proliferation determined by CFSE dilution gated on IFNγ^+^ CD4^+^ T cells (Spike n = 10; Mem n = 11). Bars represent the mean of the data set. Doughnut charts indicate fraction of donors with response to AVS (red). Response defined as *de novo* or increased cytokine production/CD154 expression. P values determined by Wilcoxon matched-pairs signed rank test.

Given the potential of AVS to exert immunomodulatory as well as antiviral activity, we examined the effect of AVS on SARS-CoV-2-specific T cell function. Analysing a publicly available single cell (sc) RNAseq data set [59] showed comparable *SOAT1* expression in CD4^+^ and CD8^+^ T cells during acute SARS-CoV-2 infection compared to healthy controls (**S3 Fig**). As expected *SOAT2* was not detected in T cells, as we previously reported [60]. PBMC isolated from the blood of unvaccinated patients hospitalised during the first pandemic wave in the UK (March-July 2020) were collected during PCR-confirmed SARS-CoV-2 infection (information about patient cohort in methods). PBMC were stimulated with peptide pools derived from SARS-CoV-2 encoded spike and membrane proteins in the presence or absence of AVS. After short-term 8 day culture, we measured key antiviral effector functions of antigen-specific CD4^+^ and CD8^+^ T cells by multiparameter flow cytometry. We identified SARS-CoV-2 spike and membrane-specific cytokine production that was significantly above the background seen in PBMC from the same donors without peptide stimulation (**S4A and 4B Figs**). AVS increased the frequency of CD4^+^ T cells producing the antiviral cytokines IFNγ, TNF (or both) and MIP1β in response to either spike or membrane peptides, boosting responses in some patients and inducing *de novo* responses in others (**Figs 6A–6C, S4C, and S4D**). The response to AVS was heterogeneous, showing a 50-fold increase in the magnitude of IFNγ-producing T cells in one patient and decreased cytokine production in a minority of patients, as previously reported for other *in vitro* and *in vivo* immunotherapeutic approaches [61,62]. A similar enhancement was seen for cytokine-producing CD8^+^ T cells in individual donors but was less consistent than for CD4^+^ T cells, resulting in non-significant changes for CD8^+^ T cell responses across the cohort (**S4E–S4G Fig**). CD4^+^ T cells provide help to activate and differentiate B cells, for example via the interaction of CD40 and CD40L (CD154). AVS increased the SARS-CoV-2-specific expression of CD154 (CD40L) on CD4^+^ T cells, reflecting an enhanced capacity to co-stimulate CD40 to activate B cells (**Fig 6D**). Consistent with the expansion of functional responses, AVS increased the proliferation of virus-specific CD4^+^ T cells (detected by CFSE dilution, **Fig 6E**).

Immunomodulatory therapies for viral infections carry the risk of increasing bystander immune responses and cytotoxic tissue damage; however, we did not detect any significant increase of CD107a mobilization to the cell membrane of perforin-producing T cells, markers of degranulation and cytotoxicity, respectively (**S4H Fig**). COVID-19 severity is associated with male sex [63] and increased age [64]. We noted that AVS enhancement of SARS-CoV-2-specific T cell responses was seen in both males and females and was independent of age (**S4I Fig**), showing the potential of this therapeutic approach for a variety of patients, including those at risk of severe infection.

To ascertain whether AVS only boosts virus-specific effector and early memory T cells during or shortly after infection but not memory T cells, we analysed a second cohort of unvaccinated donors 6 months after SARS-CoV-2 infection (memory cohort from COVIDsortium, see methods section). AVS had no consistent effect on SARS-CoV-2-specific memory CD4^+^ or CD8^+^ T cell responses 6 months post-infection (**S5A–S5D Fig**). This is in line with our previous findings showing that ACAT inhibition preferentially rescues PD-1^hi^ T cells directed against chronic viral infections or tumours and not the highly functional memory responses to cytomegalovirus [42]. AVS has shown a good safety profile in phase III atherosclerosis studies [65] and has not been associated with autoimmune responses in murine models [43]. In line with this, we did not detect any non-specific increase in cytokine production when T cells from the acute cohort were treated with AVS without viral peptides (**S5E Fig**). Thus, our data support AVS selectively expanding acutely activated SARS-CoV-2-specific T cells, without affecting memory or non-activated T cells.

## Discussion

This study raises several areas for future investigation. Our data are consistent with ACAT regulating cholesterol levels at both the cell surface and within endosomes, highlighting the need to better understand the role of cholesterol in endosomal pathways essential to virus internalisation and egress [19]. Our observation that AVS inhibited VSV-Gpp infection suggests a potential role in regulating the entry of other viruses worth further investigation. Cholesterol 25-hydroxylase catalyses the formation of 25-hydroxycholesterol (25HC) from cholesterol and leads to a redistribution of cholesterol, limiting the entry of several enveloped viruses [66] including SARS-CoV-2 [67–69]. Wang *et al* reported that 25HC activated ACAT and suggested this as a mechanism to explain 25HC inhibition of SARS-CoV-2 entry [67]. The authors showed that inhibition of ACAT with SZ58-035 partially reversed the antiviral activity of 25HC in Calu-3 cells; however, they observed a negligible effect on basal plasma membrane cholesterol levels or on SARS-CoV-2pp entry. This contrasts with our results and may reflect differences in the efficacy of SZ58-035 and AVS to modulate cholesterol levels. Furthermore, our knockdown and overexpression studies highlight a role for ACAT2 in regulating cell susceptibility to SARS-CoV-2 infection.

Our findings that ACAT inhibition altered the membrane localisation of ACE2, promoting its association with GM1 clusters and increasing the size of lipid rafts, highlights how manipulation of the intracellular esterified cholesterol content can impact the composition of the cellular membrane. An increasing body of literature shows ACE2 association with ordered (GM1) and disordered (PIP_2_) lipid regions [23,25,70], with the former promoting endosomal entry of SARS-CoV-2 and the latter augmenting spike-mediated membrane fusion at the cell surface [27]. Our pseudoparticle and viral attachment data show that inhibition of ACAT limits SARS-CoV-2 attachment and entry, potentially through dysregulated ACE2 expression on the cell surface or through disruption of factors involved in viral attachment such as heparan-sulphate, which was reported to play a role in the attachment of other coronaviruses such as infectious bronchitis virus, SARS-CoV-1 and mouse hepatitis virus [71–73]. Additionally, we cannot rule out perturbation of downstream ACAT signalling events that may modulate other factors important for viral entry yet to be defined. Further studies are required to characterise the implications of cholesterol esterification by ACAT in the context of SARS-CoV-2 attachment and entry.

Discriminating drug effects on viral entry and replication can be challenging when a virus has a short life cycle of 6-7h, as reported for SARS-CoV-2 by us and others [74,75]. Use of viral replicons that lack crucial structural proteins enables research into the formation and activity of replication complexes, providing a high-throughput platform to screen and identify antiviral compounds for SARS-CoV-2. By combining SARS-CoV-2 replicon technology with smFISH quantitation of viral RNA we demonstrated that ACAT inhibition limited the formation of replication complexes and reduced the RNA burden within cells. Cholesterol biosynthesis pathways have been identified to be essential for SARS-CoV-2 replication in several whole genome genetic studies [31–34], potentially in part due to their demonstrable role in DMV formation [35]. Inhibition of ACAT increases the level of free cholesterol and has been shown to increase oxysterols [56,76], derivatives of which can inhibit viral DMV formation [36]. This may provide a potential mechanism for the attenuation of viral replication through ACAT inhibition that is worthy of further study and highlights the key role of cholesterol homeostasis in productive viral replication.

Our findings that AVS induces the expansion of functional SARS-CoV-2-specific T cells demonstrates that perturbation of ACAT activity extends beyond effects on viral replication, to include immune boosting potential. We focused on T cells specific for two of the key structural proteins targeted in acute infection [77] and further studies to assess the effect of AVS on other T cell specificities, including those against non-structural viral proteins associated with abortive infection, would be of interest [7]. The potential for AVS to boost acutely activated CD4^+^ T effector and helper function even in the elderly, suggests ACAT inhibitors could be tested for their capacity to adjuvant sub-optimal vaccine responses in this vulnerable group [78] or others with waning immunity. The lack of T cell boosting in the memory phase is in line with our previous findings [42] but conceivably could also be related to the younger age of this cohort.

We have shown increased antiviral activity following *in vitro* treatment of T cells from the circulation; however immune responses at the site of disease, the lung and upper respiratory tract, are shaped by the local microenvironment and nutrient availability. The lung is enriched in cholesterol compared to blood [79] with cholesterol constituting the main neutral lipid in surfactant [80]. We previously reported that T cell boosting by ACAT inhibition is enhanced in the presence of high cholesterol. T cells isolated from cholesterol-rich liver and tumour tissues were boosted to a greater extent than those from the blood of the same donors [42]; suggesting a similar enhancement may be seen following ACAT inhibition of SARS-CoV-2-specific T cells infiltrating the infected lung. Although we had insufficient cryopreserved PBMC from these hospitalised donors for mechanistic experiments, we previously showed that ACAT inhibition can induce cholesterol redistribution and extensive metabolic reprogramming of virus-specific T cells [42]. Further studies to address the inflammatory response in severe and long COVID-19 would also be of interest. Consideration should be given to trials testing the efficacy of re-purposing ACAT inhibitors like AVS, an oral agent that has been shown to have a good safety profile. We show it has the capacity to exert a multipronged effect, directly inhibiting SARS-CoV-2 entry and RNA replication as well as boosting the acute T cell response that can aid viral elimination and protect against re-infection.

## Methods

### Ethics statement

The COVIDsortium cohort was approved by the ethical committee of UK National Research Ethics Service (20/SC/0149) and registered at https://ClinicalTrials.gov (NCT04318314). The Royal Free Biobank tissue access for patient benefit (TapB) was approved by the Wales Research Ethics Committee (16/WA/0289; 21/WA/0388; project approval reference: NC2020.11). The Primary Bronchial Epithelial Cell study was reviewed by the Oxford Research Ethics Committee B (18/SC/0361). All study participants gave written informed consent prior to inclusion in the study and all storage of samples obtained complied with the Human Tissue Act 2004.

### SARS-CoV-2 pseudoparticle genesis and infection

Lentiviral pseudoparticles were generated by transfecting 293T cells with p8.91 (Gag-pol), pCSFW (luciferase reporter) and a codon optimised expression construct pcDNA3.1-SARS-CoV-2-Spike, as previously described [81]. Delta and Omicron Spike expression plasmids were provided by G2P-UK National Virology consortium. Supernatants containing viral pseudotypes were harvested at 48 and 72h post-transfection. Viral titres were determined by infecting Calu-3 cells with a serial dilution of virus and 48h later measuring cellular luciferase. As a control for non-specific lentivirus uptake, stocks were generated with no envelope glycoprotein (No Env). This control was included in all experiments and luciferase values obtained subtracted from values acquired with the SARS-CoV-2pp. As an additional control pseudotypes were incubated with anti-S mAb F1-3A (1μg/mL) or ACE2-Fc (1μg/mL) for 30min prior to infection. Cell viability was assessed using a WST-8 Cell Proliferation Kit (Cayman Chemical) according to the manufacturer’s instructions.

### SARS-CoV-2 propagation and infection

Naïve VeroE6 cells were infected with SARS-CoV-2 at an MOI of 0.003 and incubated for 48-72h until visible cytopathic effect was observed. At this point, cultures were harvested, clarified by centrifugation to remove residual cell debris, and stored at -80°C. Viral titre was determined by plaque assay. Briefly, VeroE6 cells were inoculated with serial dilutions of SARS-CoV-2 viral stocks for 2h followed by addition of a semi-solid overlay consisting of 1.5% carboxymethyl cellulose (Sigma-Aldrich). Cells were incubated for 72h, visible plaques enumerated by fixing cells using amido black stain and plaque-forming units (PFU) per mL calculated. For infection of Calu-3 cells with SARS-CoV-2, cells were plated 24h before infection with the stated MOI. Cells were inoculated for 2h after which the residual inoculum was removed with three PBS washes. Unless otherwise stated, infected cells were maintained for 24h before harvesting for downstream applications. Drug treatments were applied as described in individual figure legends, for pre-infection assays drugs were applied to cells before infection and maintained throughout the duration of the experiment.

### qPCR quantification

Total cellular RNA was extracted using the RNeasy kit (Qiagen) or Trizol (Thermofisher) according to manufacturer’s instructions. For quantification of viral or cellular RNA, equal amounts of RNA, as determined by nanodrop, were used in a one-step RT-qPCR using the Takyon-One Step RT probe mastermix (Eurogentec) and run on a Roche Light Cycler 96. For quantification of viral copy numbers, qPCR runs contained serial dilutions of viral RNA standards. Total SARS-CoV-2 RNA was quantified using: 2019-nCoV_N1-F: 5’-GAC CCC AAA ATC AGC GAA AT-3’, 2019-nCoV_N1-R: 5’-TCT GGT TAC TGC CAG TTG AA TCT G-3’, 2019-nCoV_N1-Probe: 5’-FAM-ACC CCG CAT TAC GTT TGG TGG ACC-BHQ1-3’. *SOAT1* and *SOAT2* RNA was quantified by SYBR green (PCR Biosystems) using the following primers, *SOAT1_F*: 5’-GCT CGT GTT CTG GTC CTA TGT G-3’, *SOAT1_R*: 5’-TAG AAC ATC CTG TCA CCA AAG CG-3’, *SOAT2_F*: 5’-CAA TGG ACC CGA CAC ATG GA-3’, *SOAT2_R*: 5’-GGG GCA GAG GTT TGT CTT GT-3’. These were expressed relative to the housekeeper gene Beta-2-Microglobin using *B2M_F*: 5’-CTA CAC TGA ATT CAC CCC CAC TG-3’ and *B2M_R*: 5’-ACC TCC ATG ATG CTG CTT ACA TG-3’.

### Cholesterol measurement

To measure the relative changes in plasma membrane cholesterol after treatment with AVS, we developed an Amplex Red-based cholesterol detection assay. Briefly, VeroE6 cells were seeded into 96-well flat culture plates with transparent bottoms to reach confluency (~ 5 x 10^4^ per well). Cells were incubated with fresh EMEM +10%FBS for 1h followed by 1h of incubation in 100μL EMEM +10%FBS with 5μM AVS or equivalent concentrations of DMSO. After washing with 200μL PBS, cholesterol assay reactions were promptly begun by adding 100μL of working solution containing 50μM Amplex red, 1U/mL horseradish peroxidase, 2U/mL cholesterol oxidase and 2U/mL cholesterol esterase (for total cholesterol) in PBS. Relative cholesterol concentration and the background (no cells) was determined in triplicates for each sample by measuring fluorescence activity with a fluorescence microplate reader (Tecan Infinite 200 PRO, reading from bottom) for 2h at 37°C with excitation wavelength of 530nm and an emission wavelength of 585nm. Subsequently, cholesterol level was normalized to the control activity after subtracting the background.

### dSTORM imaging of GM1

VeroE6 cells were grown to 30% confluence in EMEM +10%FBS. Cells were incubated with fresh EMEM +10%FBS for 1h followed by 1h of incubation in 100μL EMEM +10%FBS with 5μM AVS or equivalent concentrations of DMSO. Cells were rinsed with PBS and then fixed with 3% paraformaldehyde and 0.1% glutaraldehyde for 15min to fix both proteins and lipids. Fixative chemicals were reduced by incubating with 0.1% NaBH4 for 7min with shaking followed by three times 10min washes with PBS. Cells were permeabilized with 0.2% Triton X-100 for 15min and then blocked with a standard blocking buffer (10% bovine serum albumin (BSA) / 0.05% Triton in PBS) for 90min at room temperature. For labelling, cells were incubated with Alexa Fluor 647-CTB (Sigma-Aldrich) for 60min in 5% BSA / 0.05% Triton / PBS at room temperature followed by 5 washes with 1% BSA / 0.05% Triton / PBS for 15min each. Cells were then washed with PBS for 5min. Cell labelling and washing steps were performed while shaking. Labelled cells were then post-fixed with fixing solution, as above, for 10min without shaking followed by three 5min washes with PBS and two 3min washes with deionized distilled water. Images were recorded with a Bruker Vutara 352 with a 60X Olympus Silicone objective. Frames with an exposure time of 20ms were collected for each acquisition. Excitation of the Alexa Fluor 647 dye was achieved using 640nm lasers and Cy3B was achieved using 561nm lasers. Laser power was set to provide isolated blinking of individual fluorophores. Cells were imaged in a photo-switching buffer comprising of 1% β-mercaptoethanol (Sigma-Aldrich), oxygen scavengers (glucose oxidase and catalase; (Sigma-Aldrich) in 50mM Tris (Affymetrix) +10mM NaCl (Sigma-Aldrich) +10% glucose (Sigma) at pH 8.0. Axial sample drift was corrected during acquisition through the Vutara 352’s vFocus system. Images were constructed using the default modules in the Zen software. Each detected event was fitted to a 2D Gaussian distribution to determine the centre of each point spread function plus the localization precision. Pair correlation and cluster analysis was performed using the Statistical Analysis package in the Vutara SRX software. Pair correlation analysis is a statistical method used to determine the strength of correlation between two objects by counting the number of points of probe 2 within a certain donut-radius of each point of probe 1. This allows for localization to be determined without overlapping pixels as done in traditional diffraction-limited microscopy. Cluster size estimation and cluster density were calculated through cluster analysis by measuring the length and density of the clusters comprising of more than 10 particles with a maximum particle distance of 0.1μm.

### SARS-CoV-2 replicon assays

A SARS-CoV-2 replicon (pSC2-Rep-Del-p-RL-7NG) corresponding to the Delta Variant of Concern (VOC) was used. The coding regions for the Spike, membrane and ORF7a proteins were replaced with those of puromycin N-acetyl transferase, *Renilla* luciferase and mNeonGreen respectively [53]. Replicon RNA transcripts and an N gene cDNA template were generated with T7 polymerase using a RiboMAX Large Scale RNA Production System-T7 (Promega) and capped using anti-reverse cap analogue (NEB). RNA was precipitated with lithium chloride and resuspended in ultra-pure water. VeroE6-ACE2-TMPRSS2 cells [82]; were co-transfected using a Neon Transfection System (Invitrogen, ThermoFisher) with 2μg N-gene mRNA and 10μg replicon genomic RNA. 50,000 cells were seeded onto poly-D-lysine coated coverslips and treated with AVS (10μM), RDV (1μM) or control DMSO in Dulbecco’s Modified Eagle’s medium, containing 4.5g/l D-glucose, and GlutaMAX (Gibco, ThermoFisher) supplemented with 1mM sodium pyruvate (Sigma-Aldrich), 10% (v/v) foetal bovine serum (Gibco, ThermoFisher) and penicillin (50 units/mL)/streptomycin (50 μg/mL) at 37°C in a humidified incubator in 5% CO_2_. At an appropriate time post-transfection the cells were fixed with 4% (v/v) paraformaldehyde in PBS for 15min. Coverslips were then washed, and coverslips stored in PBS containing 0.1% (v/v) diethylpyrocarbonate until processing for smFISH.

### smFISH of SARS-CoV-2 RNA

smFISH was carried out as previously reported [54]. Briefly, Vero-TMPRSS2 cells were grown on #1.5 round-glass coverslips in a 24-well plate, fixed in 4% paraformaldehyde (Thermo Fisher) for 30min at room temperature. Cells were permeabilised in PBS/0.1% Triton X-100 for 10min at room temperature followed by washes in PBS and 2X SSC. Cells were pre-hybridised in pre-warmed (37°C) wash solution (2× SSC, 10% formamide) twice for 20min at 37°C. smFISH probes were diluted to 500nM in hybridisation solution (2× SSC, 10% formamide, 10% dextran sulphate) and incubated overnight at 37°C. Coverslips were then washed for 20min in pre-warmed wash solution at 37°C followed by counterstain with DAPI (1μg/mL) and CellMask Green (1:1,000,000) diluted in wash solution. Cells were washed once with wash solution for 20min at 37°C and twice with 2× SSC for 10min at room temperature. Coverslips were mounted using Vectashield onto glass slides and imaged on an Olympus SoRA Super-res confocal system. Automatic and manual image acquisition and image stitching were performed with Olympus cellSens Dimension software. Images were analysed with a custom Python pipeline using Bigfish, skimage, and numpy libraries (available in the GitHub repository https://github.com/jefflee1103/Lee_Wing-SARS2/tree/main/smFISH/analaysis-codes/Bigfish-pipeline). Tiff files were converted to a numpy array, and individual cells segmented from the image using the Cellpose library. Background signal in the smFISH channel was subtracted with the skimage.white_tophat algorithm (radius = 5, individual z frames were processed in 2D due to memory constraints, results were indistinguishable from 3D-processed images). Threshold setting for smFISH spot detection was set specifically for each set of images collected in each session. Replication foci were defined as gRNA smFISH signals with spatially extended foci that exceed the point-spread function of the microscope and intensity of the reference single molecules.

### SARS-CoV-2 attachment assay

Vero-TMPRSS2 cells were pre-incubated with 10μM AVS, 1μg/mL Anti-spike mAb FI-3A, 100U/mL Heparin or 1μg/mL ACE2-Fc for 2h at 37°C. Cells were then infected with SARS-CoV-2 (MOI = 10) at 4°C for 2h, unbound virus was removed by extensive washing in PBS, and samples fixed in 4% paraformaldehyde. For immunofluorescent staining of the viral nucleocapsid cells were permeabilised with PBS 0.1% Triton X-100 for 30min at room temperature. Samples were blocked in PBS 3% bovine serum albumin (BSA) for 1h room temperature. Cells were incubated with 2μg/mL of the human anti-N primary antibody (EY-2A clone) [52] overnight at 4°C. Cells were washed three times in PBS/0.1% Tween-20 for 10min each at room temperature and incubated with fluorescent secondary antibodies (1:1000) diluted in blocking solution for 1h at room temperature. After a further three washes in PBS, cells were mounted using Vectashield and imaged as above.

### Western blot

Cells were washed with PBS and lysed using RIPA buffer (20 mM Tris, pH 7.5, 2mM EDTA, 150 mM NaCl, 1% NP40, and 1% sodium deoxycholate) supplemented with protease inhibitor cocktail tablets (Roche). Clarified samples were mixed with Laemmli sample buffer, separated by SDS-PAGE and proteins transferred to polyvinylidene difluoride membrane. Membranes were blocked in 5% milk in PBS/0.1% Tween-20 and incubated with anti-TMPRSS2 (Proteintech Europe Ltd), anti-β-Actin (Sigma), anti-SOAT1 (cell signalling) or anti-SOAT2 (abcam) and appropriate HRP-conjugated secondary antibodies (DAKO). Chemiluminescence substrate (West Dura, 34076, Thermo Fisher Scientific) was used to visualize proteins using a G:Box Imaging system (Syngene).

### siRNA transfection

Calu-3 or VeroE6-TMPRSS2 were reverse transfected with 50nM of siRNAs directed against *SOAT1*, *SOAT2* or a scramble control (Ambion) complexed with DharmaFECT4 transfection reagent (Dharmacon) according to manufacturer’s instructions. Transfected cells were seeded and left to recover for 4h after which media was replenished. Cells were infected with a SARS-CoV-2 mNeonGreen reporter virus at an MOI of 0.01 24h post siRNA delivery. Viral replication was assessed through quantification of mNeonGreen fluorescence using a ClarioStar plate reader (BMG-LABTECH). Data was normalised to DAPI staining to account for cell density.

### ACAT overexpression

VeroE6-TMPRSS2 cells were transfected with 1μg of pBABE-puro constructs containing either wild-type or catalytically inactive mutant ACAT1 (H460A) or ACAT2 (H360A) respectively using Fugene transfection reagent (Promega). Cells were infected with the SARS-CoV-2-mNeonGreen reporter virus and replication quantified as above.

### Patient cohort

Peripheral blood samples were taken from unvaccinated study participants during or after SARS-CoV-2 infection during the first pandemic wave of infections in the UK (March-July 2020). The Acute Cohort was recruited from hospitalized patients at the Royal Free Hospital, London, and SARS-CoV-2 infection was confirmed by PCR (n = 22; median age 82 years; 45% female, 55% male; 73% white, 4% black, 14% Asian, 9% other). The Memory Cohort (COVIDsortium) was recruited from healthcare workers in London and SARS-CoV-2 infection was confirmed by PCR and/or serology. Samples were taken 5–6 months post infection (n = 12; median age 44.5 years; 50% female, 50% male; 50% white, 8% black, 34% Asian, 8% other). More information about COVIDsortium can be found in [83].

### PBMC isolation

For samples taken during acute SARS-CoV-2 infection, PBMC were isolated from EDTA blood using Histopaque-1077 (Sigma-Aldrich) density-gradient centrifugation in Leukosep tubes (Greiner Bio One) according to the manufacturer’s instructions. For the COVIDsortium cohort, PBMC were isolated from heparinized blood samples using Pancoll (Pan Biotech) or Histopaque-1077 Hybri-Max (Sigma-Aldrich) density-gradient centrifugation in SepMate tubes (StemCell) according to the manufacturer’s instructions. Isolated PBMC were cryopreserved in fetal bovine serum (FBS; Sigma-Aldrich) containing 10% dimethyl sulfoxide (DMSO; Sigma-Aldrich) and stored in liquid nitrogen prior to cell culture.

### Short-term cell culture

To examine SARS-CoV-2-specific T cell responses in the blood, PBMC were stimulated with 1μg/mL SARS-CoV-2 peptide pools (Membrane (Mem): 15mer peptides overlapping by 10aa, 43 peptides total; Spike S1: 18-20mer peptides, 18 peptides total. The full peptide sequences can be found in [84]) in cRPMI (RPMI 1640 (Thermo Fisher Scientific) +2% HEPES buffer solution, 0.5% sodium pyruvate, 0.1% 2-mercaptoethanol, MEM 1% non-essential and 2% essential amino acids; Gibco, and 100U/mL penicillin/streptomycin; life technologies) +10% FBS +20U/mL recombinant human IL-2 (PeproTech)+ 5μg/mL anti-CD28 (Invitrogen). PBMC were expanded at 37°C for 8 days ± 0.5μM of the ACAT inhibitor Avasimibe (AVS; Selleckchem) or equivalent concentration of DMSO replenished every 2d. On day 7, PBMC were restimulated with 1μg/mL peptide +anti-CD28 in the presence of 1μg/mL Brefeldin A (Sigma-Aldrich) for 16h at 37°C, followed by antibody staining and flow cytometric analysis. All cultures were performed in duplicate wells that were combined prior to restimulation. Post-culture viability of PBMC was confirmed and samples with <50% viable cells were excluded from further analysis. In all summary data, the cytokine production/CD154 expression in the condition without peptides (using PBMC from the same donor and time point) was subtracted from the cytokine production/CD154 expression in the peptide-stimulated wells to determine SARS-CoV-2-specific cytokine production/CD154 expression. A SARS-CoV-2-specific response was defined as a minimum of 10 cells in the positive fraction. For evaluation of cell proliferation, PBMCs were labelled with 1μM CFDA-SE (Thermo Fisher) prior to the start of culture.

### Surface and intracellular staining

For flow cytometry, cells were stained with saturating concentrations of surface antibodies and a fixable viability dye diluted in 1:1 PBS (Invitrogen): Brilliant Violet Buffer (BD Biosciences). Following surface staining, cells were fixed and permeabilized with cytofix/cytoperm (BD Biosciences) followed by an intracellular staining with antibodies in saturating concentrations diluted in a 0.1% saponin-based buffer (Sigma-Aldrich). Full details on fluorescent monoclonal antibodies can be found in S1 Table. All samples were acquired on a BD Biosciences Fortessa-X20 or Fortessa and analysed using FlowJo v.10 (BD Biosciences).

### Human PBEC

Biopsies were obtained using flexible fibreoptic bronchoscopy from healthy control volunteers under light sedation with fentanyl and midazolam. Airway epithelial cells were taken using 2mm diameter cytology brushes from 3^rd^ to 5^th^ order bronchi and cultured in Airway Epithelial Cell medium (PromoCell) in submerged culture.

### scRNA-seq data analysis

Processed count matrices with de-identified metadata were downloaded from https://www.covid19cellatlas.org/index.patient.html (raw data also available at Expression Omnibus, GSE150728). All data processing steps are described in Wilk et. al, [59] including: pre- and post-alignment quality control, scaling, transformation, normalisation, clustering, and dimension reduction. The normalised expression levels of ACAT1 and ACAT2 taken from processed data was plotted by cell subset using “cell.type.coarse”.

### Statistical analysis

Statistical analyses were performed with Prism 7.0 (GraphPad) as indicated in figure legends (Wilcoxon matched-pairs signed-rank test, Mann–Whitney test, Spearman correlation, Kruskall Wallis, unpaired t test) with significant differences marked on all figures. In experiments with a sample size >100 normality was assessed using a D’Agostino-Pearson omnibus normality test. All tests were performed as two-tailed tests, and for all tests, significance levels were defined as not significant (ns) P ≥ 0.05; *P < 0.05; **P < 0.01; ***P < 0.001; ****P < 0.0001.

## Supporting information

S1 Fig Expression of TMPRSS2 in Vero cells and effect of Avasimibe on the pseudoparticle luciferase reporter gene expression and cholesterol levels. (a) Immunoblot of TMPRSS2 in parental Vero and Vero-TMPRSS2 cells. (b) Vero-TMPRSS2 cells were transfected with the luciferase reporter construct used to generate SARS-CoV-2 Spike pseudo-particles and treated with 10μM of AVS. Luciferase activity was quantified 24h post infection and expressed as relative light units per μg of total cellular protein. (c) Free and total cholesterol levels in VeroE6 cells treated with 5μM Avasimibe (AVS) or DMSO for 60min. Data normalised to mean of DMSO control (n = 4).
(PDF)

S2 Fig Expression of *SOAT*1/2 and effect of Avasimibe and Nevanimibe on SARS-CoV-2 replication. (a) VeroE6-TMPRSS2 cells were infected with SARS-CoV-2 (MOI 0.01) for 2h, the inoculum removed, and cells treated with AVS or 1μM of RDV. Cells were harvested 24h post infection and intracellular viral RNA quantified by qPCR. Data are representative of n = 4 biological replicates. Statistical significance was determined by ANOVA (Kruskal-Wallis). (b) qPCR quantification of *SOAT1* and *SOAT2* in Vero-TMPRSS2, Calu-3 and PBEC. Data are the delta Ct between *SOAT1* or *SOAT2* and Beta-2-Microglobin. (c) FPKM read counts of *Soat1* and *Soat2* from RNAseq data of SARS-CoV-2 infected Golden-Syrian hamster lung samples (n = 5). (d) Western blot of ACAT1 and ACAT2 expression in Vero and Vero-TMPRSS2. (e) VeroE6-TMPRSS2 were infected with SARS-CoV-2_mNeonGreen at an MOI of 0.01 and treated with increasing doses of AVS, Nevanimibe and RDV. Viral replication was assessed by measuring fluorescence and expressed relative to the DMSO control.
(PDF)

S3 Fig ACAT1 and ACAT2 expression in PBMC. Expression of ACAT1 and ACAT2 by cell subset in PBMC taken 2–16 days following symptom onset from donors with severe acute SARS-CoV-2 infection (n = 7) or from healthy control is shown (n = 6). Analysis of publicly available scRNA-seq data from Wilk et al [59]. The average expression of ACAT1 and ACAT2 by cell subset is shown in the table below. No significant differences in ACAT1 or ACAT2 expression was seen between cohorts (Wilcoxon rank-sum test).
(PDF)

S4 Fig Effect of Avasimibe on T cell function in acute SARS-CoV-2 infection. (a) Flow cytometry gating strategy for PBMC (lymphocytes, single cells, alive, CD3^+^, CD4^+^/CD8^-^ or CD4^-^/CD8^+^). (b-i) Human PBMC from donors with acute SARS-CoV-2 infection were stimulated with SARS-CoV-2 peptide pools (Spike and Membrane, Mem) for 8 days and analysed via flow cytometry. (b) IFNγ production by CD8^+^ and CD4^+^ T cells without peptide and after stimulation with Spike and Mem peptide pools. (c-i) PBMC were stimulated with Spike and Mem peptide pools and treated with AVS or DMSO for 8 days and analysed via flow cytometry. The cytokine production in wells without peptide stimulation was subtracted to determine SARS-CoV-2-specific cytokine production in summary data. (c) Fold change of IFNγ production by CD4^+^ T cells with AVS compared to DMSO (n = 13). (d) SARS-CoV-2-specific IFNγ and TNF production by CD4^+^ T cells. (e-g) SARS-CoV-2 specific IFNγ (e), TNF (f), MIP1β (g) production by CD8^+^ T cells (n = 19). (h) SARS-CoV-2-specific upregulation of CD107a and production of perforin (perf) by CD4^+^ and CD8^+^ T cells (n = 11). (i) Fold change of SARS-CoV-2-specific (Spike+Mem) IFNγ production by CD4^+^ T cells from donors during acute infection assessed by sex (left) and correlated with donor age (right). Bars represent the mean of the data set. Doughnut charts indicate fraction of donors with response to AVS (red). Response defined as *de novo* or increased cytokine production. P values determined by Kruskal-Wallis test (b), Wilcoxon matched-pairs signed rank test (c-h) and Mann-Whitney (i, left). Correlation assessed by Spearman correlation (i, right).
(PDF)

S5 Fig Effect of Avasimibe on SARS-CoV-2-specific memory T cell function. (a-d) Human PBMC from donors 6 months post infection were stimulated with SARS-CoV-2 peptide pools (Spike and Membrane, Mem) and treated with Avasimibe (AVS) or DMSO for 8 day. SARS-CoV-2-specific cytokine production by T cells was detected via flow cytometry. The cytokine production and CD154 expression in wells without peptide stimulation was subtracted to determine SARS-CoV-2-specific cytokine production/CD154 expression in summary data. (a-d) SARS-CoV-2-specific IFNγ (a), TNF (b), MIP1β (c) production and CD154 expression (d) from donors 6 months post SARS-CoV-2 infection (Spike n = 12; Mem n = 11). (e) IFNγ production by T cells from donors with acute SARS-CoV-2 infection without *in vitro* peptide stimulation. Bars represent the mean of the data set. Doughnut charts indicate fraction of donors with response to AVS (red). Response defined as *de novo* or increased cytokine production/CD154 expression. P values determined by Wilcoxon matched-pairs signed rank test.
(PDF)

S1 Table Fluorescent reagents for flow cytometry. (PDF)

S1 Acknowledgements List of COVIDsortium Investigators. (PDF)
