# Supplementary figures and images for: An ACAT inhibitor suppresses SARS-CoV-2 replication and boosts antiviral T cell activity
Source: PLoS Pathog. 2023 May 3;19(5):e1011323. doi: 10.1371/journal.ppat.1011323 (PMC10202285; doi:10.1371/journal.ppat.1011323)

S1 Fig

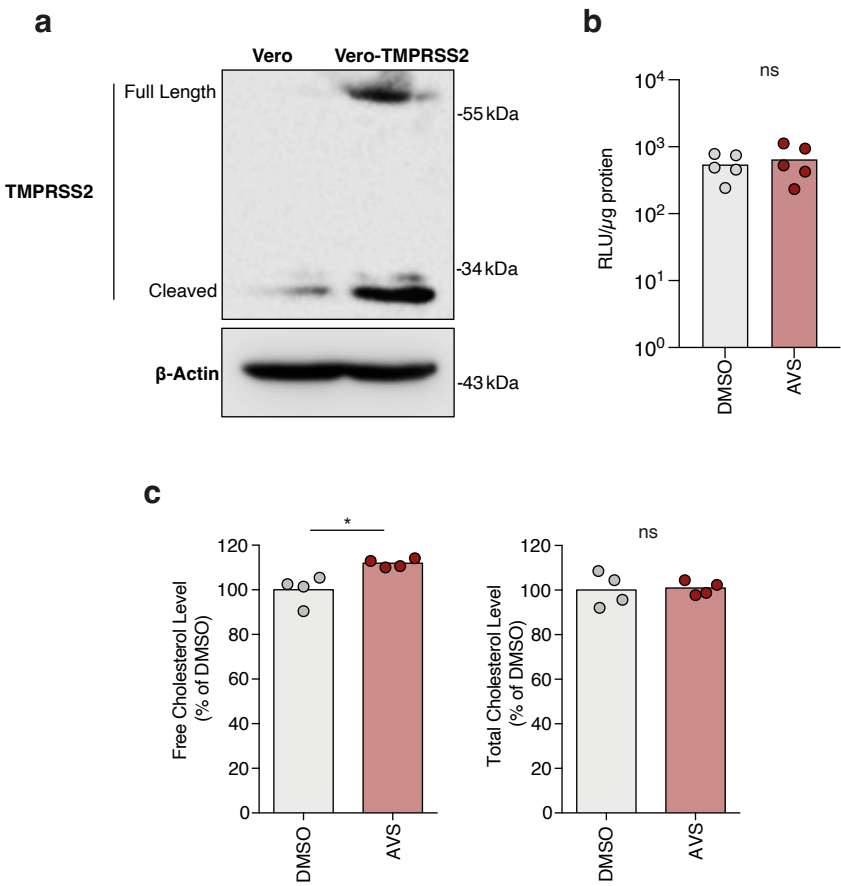

Supplement: S1 Fig — (a) Immunoblot of TMPRSS2 in parental Vero and Vero-TMPRSS2 cells. (b) Vero-TMPRSS2 cells were transfected with the luciferase reporter construct used to generate SARS-CoV-2 Spike pseudo-particles and treated with 10μM of AVS. Luciferase activity was quantified 24h post infection and expressed as relative light units per μg of total cellular protein. (c) Free and total cholesterol levels in VeroE6 cells treated with 5μM Avasimibe (AVS) or DMSO for 60min. Data normalised to mean of DMSO control (n = 4). (PDF) [file ppat.1011323.s001.pdf]

S2 Fig

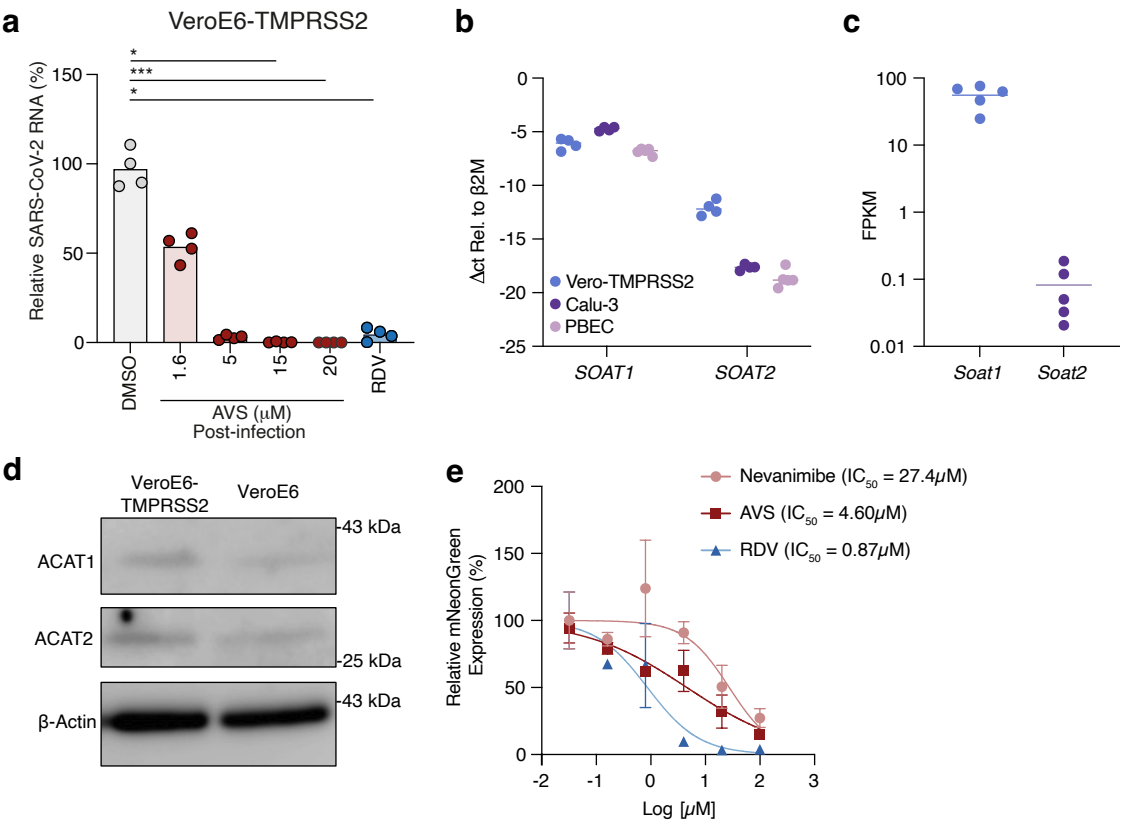

Supplement: S2 Fig — (a) VeroE6-TMPRSS2 cells were infected with SARS-CoV-2 (MOI 0.01) for 2h, the inoculum removed, and cells treated with AVS or 1μM of RDV. Cells were harvested 24h post infection and intracellular viral RNA quantified by qPCR. Data are representative of n = 4 biological replicates. Statistical significance was determined by ANOVA (Kruskal-Wallis). (b) qPCR quantification of SOAT1 and SOAT2 in Vero-TMPRSS2, Calu-3 and PBEC. Data are the delta Ct between SOAT1 or SOAT2 and Beta-2-Microglobin. (c) FPKM read counts of Soat1 and Soat2 from RNAseq data of SARS-CoV-2 infected Golden-Syrian hamster lung samples (n = 5). (d) Western blot of ACAT1 and ACAT2 expression in Vero and Vero-TMPRSS2. (e) VeroE6-TMPRSS2 were infected with SARS-CoV-2_mNeonGreen at an MOI of 0.01 and treated with increasing doses of AVS, Nevanimibe and RDV. Viral replication was assessed by measuring fluorescence and expressed relative to the DMSO control. (PDF) [file ppat.1011323.s002.pdf]

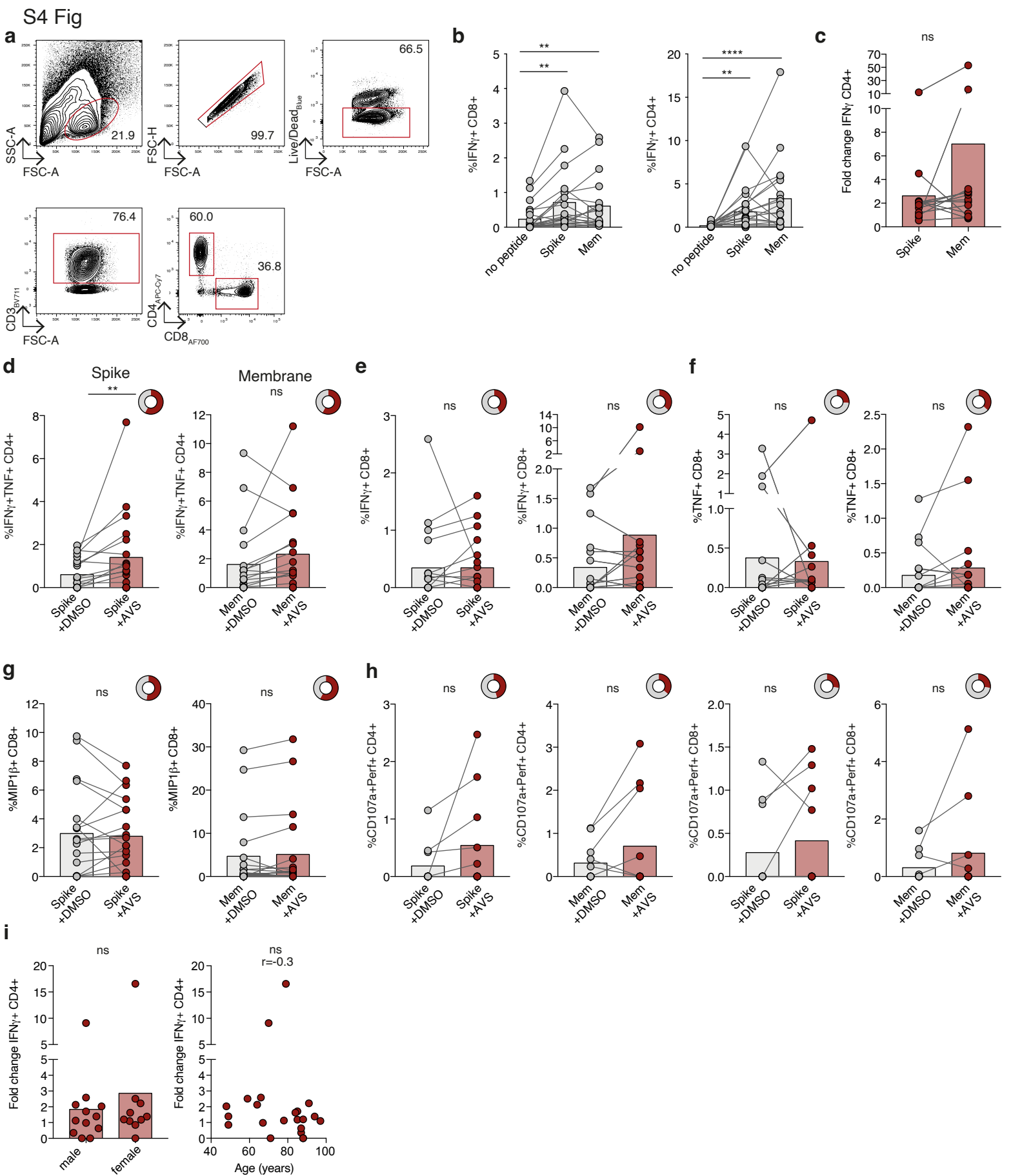

Supplement: S4 Fig — (a) Flow cytometry gating strategy for PBMC (lymphocytes, single cells, alive, CD3+, CD4+/CD8- or CD4-/CD8+). (b-i) Human PBMC from donors with acute SARS-CoV-2 infection were stimulated with SARS-CoV-2 peptide pools (Spike and Membrane, Mem) for 8 days and analysed via flow cytometry. (b) IFNγ production by CD8+ and CD4+ T cells without peptide and after stimulation with Spike and Mem peptide pools. (c-i) PBMC were stimulated with Spike and Mem peptide pools and treated with AVS or DMSO for 8 days and analysed via flow cytometry. The cytokine production in wells without peptide stimulation was subtracted to determine SARS-CoV-2-specific cytokine production in summary data. (c) Fold change of IFNγ production by CD4+ T cells with AVS compared to DMSO (n = 13). (d) SARS-CoV-2-specific IFNγ and TNF production by CD4+ T cells. (e-g) SARS-CoV-2 specific IFNγ (e), TNF (f), MIP1β (g) production by CD8+ T cells (n = 19). (h) SARS-CoV-2-specific upregulation of CD107a and production of perforin (perf) by CD4+ and CD8+ T cells (n = 11). (i) Fold change of SARS-CoV-2-specific (Spike+Mem) IFNγ production by CD4+ T cells from donors during acute infection assessed by sex (left) and correlated with donor age (right). Bars represent the mean of the data set. Doughnut charts indicate fraction of donors with response to AVS (red). Response defined as de novo or increased cytokine production. P values determined by Kruskal-Wallis test (b), Wilcoxon matched-pairs signed rank test (c-h) and Mann-Whitney (i, left). Correlation assessed by Spearman correlation (i, right). (PDF) [file ppat.1011323.s004.pdf]

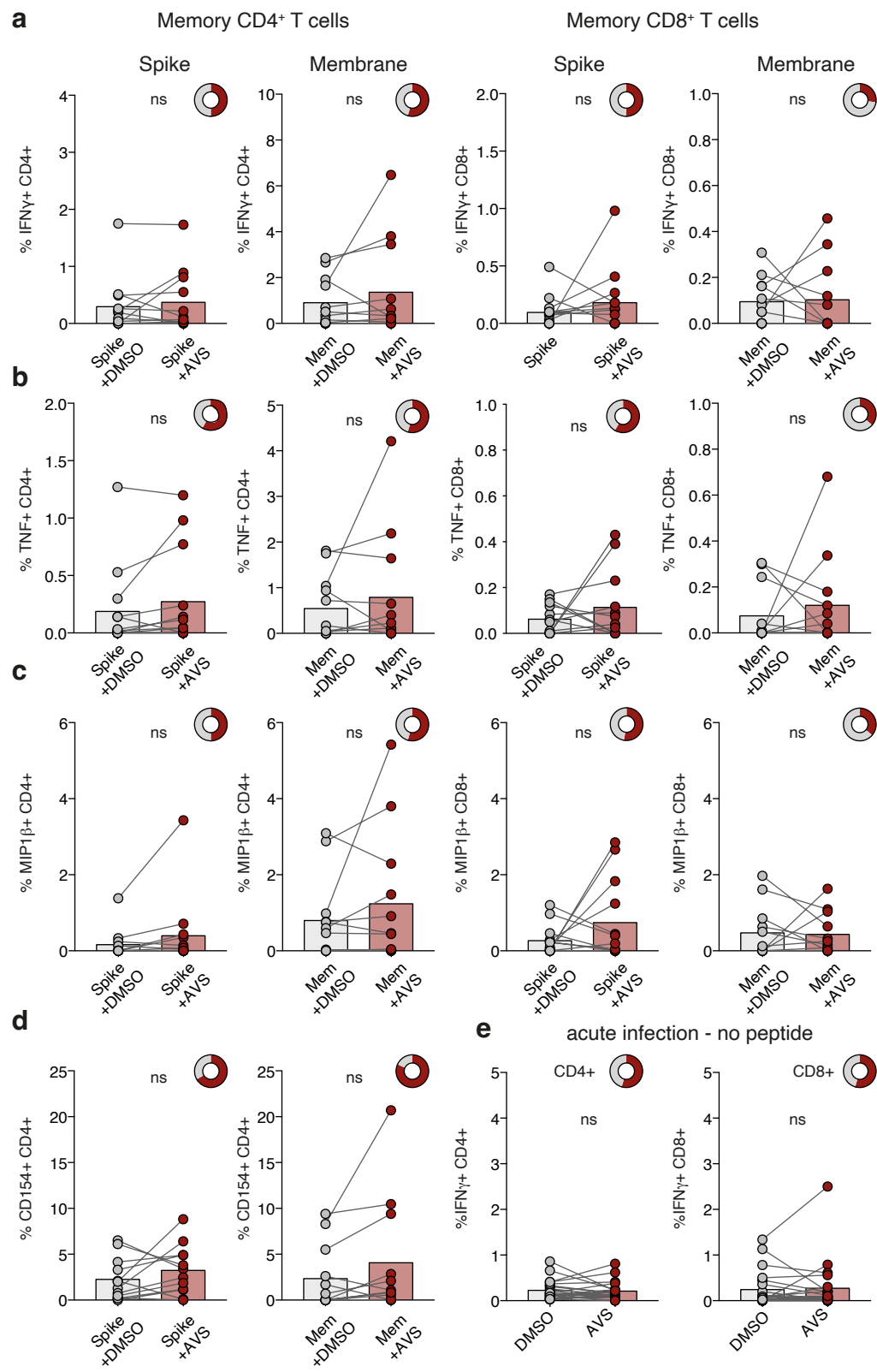

Supplement: S5 Fig — (a-d) Human PBMC from donors 6 months post infection were stimulated with SARS-CoV-2 peptide pools (Spike and Membrane, Mem) and treated with Avasimibe (AVS) or DMSO for 8 day. SARS-CoV-2-specific cytokine production by T cells was detected via flow cytometry. The cytokine production and CD154 expression in wells without peptide stimulation was subtracted to determine SARS-CoV-2-specific cytokine production/CD154 expression in summary data. (a-d) SARS-CoV-2-specific IFNγ (a), TNF (b), MIP1β (c) production and CD154 expression (d) from donors 6 months post SARS-CoV-2 infection (Spike n = 12; Mem n = 11). (e) IFNγ production by T cells from donors with acute SARS-CoV-2 infection without in vitro peptide stimulation. Bars represent the mean of the data set. Doughnut charts indicate fraction of donors with response to AVS (red). Response defined as de novo or increased cytokine production/CD154 expression. P values determined by Wilcoxon matched-pairs signed rank test. (PDF) [file ppat.1011323.s005.pdf]
