# Supplementary material for: An ACAT inhibitor suppresses SARS-CoV-2 replication and boosts antiviral T cell activity
Source: PLoS Pathog. 2023 May 3;19(5):e1011323. doi: 10.1371/journal.ppat.1011323 (PMC10202285; doi:10.1371/journal.ppat.1011323)
Supplement: S3 Fig — Expression of ACAT1 and ACAT2 by cell subset in PBMC taken 2–16 days following symptom onset from donors with severe acute SARS-CoV-2 infection (n = 7) or from healthy control is shown (n = 6). Analysis of publicly available scRNA-seq data from Wilk et al [59]. The average expression of ACAT1 and ACAT2 by cell subset is shown in the table below. No significant differences in ACAT1 or ACAT2 expression was seen between cohorts (Wilcoxon rank-sum test). (PDF) [file ppat.1011323.s003.pdf]

# S3 Fig

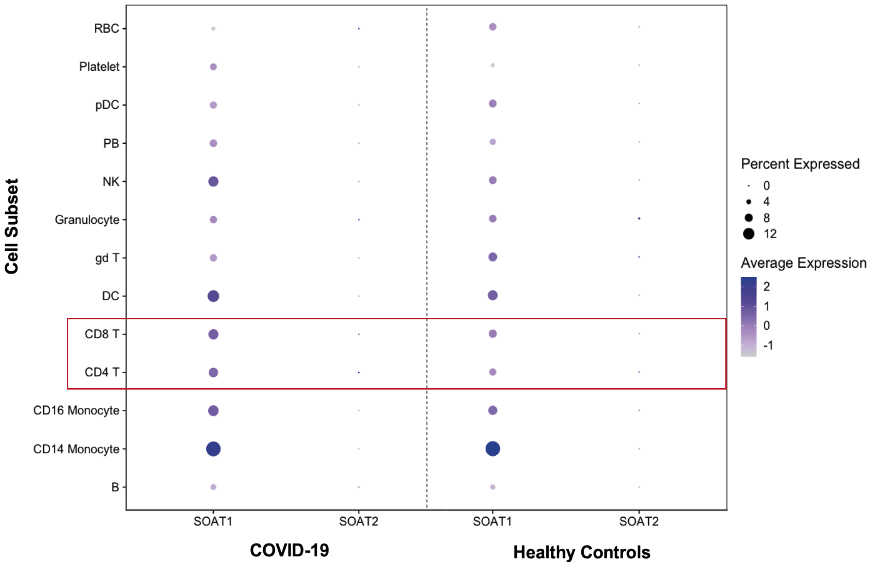


| SOAT1 | | |
|---|---|---|
| | COVID-19 | Healthy Control |
| | Average Expression | Average Expression |
| RBC | 0.03 | 0.07 |
| Platelet | 0.07 | 0.03 |
| pDC | 0.07 | 0.08 |
| PB | 0.07 | 0.06 |
| NK | 0.11 | 0.09 |
| Granulocyte | 0.07 | 0.09 |
| gd T | 0.07 | 0.10 |
| DC | 0.12 | 0.11 |
| CD8 T | 0.10 | 0.08 |
| CD4 T | 0.10 | 0.09 |
| CD16 Monocyte | 0.11 | 0.10 |
| CD14 Monocyte | 0.16 | 0.17 |
| B | 0.05 | 0.05 |

| SOAT2 | | |
|---|---|---|
| | COVID-19 | Healthy Control |
| | Average Expression | Average Expression |
| RBC | 0.00 | 0.00 |
| Platelet | 0.00 | 0.00 |
| pDC | 0.00 | 0.00 |
| PB | 0.00 | 0.00 |
| NK | 0.00 | 0.00 |
| Granulocyte | 0.00 | 0.01 |
| gd T | 0.00 | 0.00 |
| DC | 0.00 | 0.00 |
| CD8 T | 0.00 | 0.00 |
| CD4 T | 0.00 | 0.00 |
| CD16 Monocyte | 0.00 | 0.00 |
| CD14 Monocyte | 0.00 | 0.00 |
| B | 0.00 | 0.00 |
