# Supplementary material for: An ACAT inhibitor suppresses SARS-CoV-2 replication and boosts antiviral T cell activity
Source: PLoS Pathog. 2023 May 3;19(5):e1011323. doi: 10.1371/journal.ppat.1011323 (PMC10202285; doi:10.1371/journal.ppat.1011323)
Supplement: S1 Table — (PDF) [file ppat.1011323.s006.pdf]

Supplementary Table 1 Fluorescent reagents for flow cytometry

Antigen	Fluorochrome	Clone	Supplier	Catalogue number
**Monoclonal antibodies for flow cytometry**				
CD3	BUV805	UCHT1	BD Biosciences	612895
CD4	BUV395	SK3	BD Biosciences	563550
CD8	Alexa Fluor 700	RPA-T8	BioLegend	301028
CD8	BV785	RPA-T8	BioLegend	301046
CD154	PE/Cyanine7	24-31	BioLegend	310832
IFNγ	V450	B27	BD Biosciences	560371
TNF	APC/Cyanine7	MAb11	BioLegend	502944
TNF	PE/Dazzle 594	MAb11	BioLegend	502945
MIP1b	PE	D21-1351	BD Biosciences	550078
Perforin	APC/Cyanine7	dG9	BioLegend	308128
Perforin	PE/Dazzle 594	dG9	BioLegend	308132
Perforin	PerCP/Cy5.5	B-D48	BioLegend	353314
CD107a	APC	H4A3	BioLegend	328620
**Other fluorescent reagents for flow cytometry**				
Vybrant CFDA SE Cell Tracer Kit	na	na	Invitrogen	V12883
LIVE/DEAD Fixable Blue Dead Cell Stain Kit	na	na	Invitrogen	L23105
