# Supplementary material for: An ACAT inhibitor suppresses SARS-CoV-2 replication and boosts antiviral T cell activity
Source: PLoS Pathog. 2023 May 3;19(5):e1011323. doi: 10.1371/journal.ppat.1011323 (PMC10202285; doi:10.1371/journal.ppat.1011323)
Supplement: S1 Acknowledgements — (PDF) [file ppat.1011323.s007.pdf]

COVIDsortium Investigators

The members of the UK COVIDsortium investigators are Hakam Abbass, Aderonke Abiodun, Mashael Alfarih, Zoe Alldis, Daniel M Altmann, Oliver E Amin, Mervyn Andiapen, Jessica Artico, João B Augusto, Georgina L Baca, Sasha N L. Bailey, Anish N Bhuva, Alex Boulter, Ruth Bowles, Rosemary J Boyton, Olivia V Bracken, Ben O'Brien, Tim Brooks, Natalie Bullock, David K Butler, Gabriella Captur, Olivia Carr, Nicola Champion, Carmen Chan, Aneesh Chandran, Tom Coleman, David Collier, Jorge Couto de Sousa, Xose Couto-Parada, Eleanor Cross, Teresa Cutino-Moguel, Silvia D'Arcangelo, Rhodri H Davies, Brooke Douglas, Cecilia Di Genova, Keenan Dieobi-Anene, Mariana O Diniz, Anaya Ellis, Karen Feehan, Malcolm Finlay, Marianna Fontana, Nasim Forooghi, Sasha Francis, Celia Gaier, Joseph M Gibbons, Derek Gilroy, Matt Hamblin, Gabrielle Harker, Georgia Hemingway, Jacqueline Hewson, Wendy Heywood, Lauren M Hickling, Bethany Hicks, Aroon D Hingorani, Lee Howes, Alun Hughes, Gemma Hughes, Rebecca Hughes, Ivie Itua, Victor Jardim, Wing-Yiu Jason Lee, Melaniepetra Jensen, Jessica Jones, Meleri Jones, George Joy, Vikas Kapil, Caoimhe Kelly, Hibba Kurdi, Jonathan Lambourne, Kai-Min Lin, Siyi Liu, Aaron Lloyd, Sarah Louth, Mala K Maini, Vineela Mandadapu, Charlotte Manisty, Áine McKnight, Katia Menacho, Celina Mfuko, Kevin Mills, Sebastian Millward, Oliver Mitchelmore, Christopher Moon, James C Moon, Diana Muñoz Sandoval, Sam M Murray, Mahdad Noursadeghi, Ashley Otter, Corinna Pade, Susana Palma, Ruth Parker, Kush Patel, Mihaela Pawarova, Babita Pawarova, Steffen E Petersen, Brian Piniera, Franziska P Pieper, Daniel Pope, Mary Prossora, Lisa Rannigan, Alicja Rapala, Catherine J Reynolds, Amy Richards, Matthew Robathan, Joshua Rosenheim, Cathy Rowe, Matthew Royds, Jane Sackville West, Genine Sambile, Nathalie M Schmidt, Hannah Selman, Amanda Semper, Andreas Seraphim, Mihaela Simion, Angelique Smit, Michelle Sugimoto, Leo Swadling, Stephen Taylor, Nigel Temperton, Stephen Thomas, George D Thornton, Thomas A Treibel, Art Tucker, Ann Varghese, Jessry Veerapen, Mohit Vijayakumar, Tim Warner, Sophie Welch, Hannah White, Theresa Wodehouse, Lucinda Wynne, and Dan Zahedi.
